# Tracking rehabilitated sea turtles in the Indian Ocean using satellite telemetry: Insights into behaviour, ecology, and conservation implications

**DOI:** 10.1371/journal.pone.0351541

**Published:** 2026-06-26

**Authors:** Katrina Himpson, Thomas Le Berre, Edward Hodges

**Affiliations:** Reefscapers Ltd Plc, Male, Republic of the Maldives; Universidad de Cadiz Facultad de Ciencias del Mar y Ambientales, SPAIN

## Abstract

Understanding the movements and behaviour of threatened marine turtles is essential for effective conservation management. Satellite telemetry is a valuable tool which has previously provided valuable insights sea turtle behaviour, particularly mating and post-nesting movements; however, certain life stages and species remain under-represented. Here, we use satellite tracking to investigate the post-rehabilitation behaviour of olive ridley, green, and hawksbill turtles in the Indian Ocean. We find that rehabilitated turtles survive for extended periods following release and display species and life-stage specific behaviour comparable to wild individuals. Monsoon-driven ocean currents strongly influence movements in open ocean with turtles actively swimming to maintain position or navigate along continental shelves and ridges. These findings provide valuable insights into the behaviour of groups under-represented in current literature, namely olive ridley and juvenile turtles, demonstrates the value of rehabilitation for both facilitating research and supporting animal welfare, and underscores the importance of regional cooperation for the conservation of these wide-ranging species.

## Introduction

Marine turtles are an ecologically and economically important group of reptiles found across tropical, sub-tropical and temperate oceans [1–3]. Due to their delayed sexual maturity and high mortality rate in early life stages, five of the seven known species of marine turtles are considered vulnerable to extinction [2]. A further species remains unclassified through lack of substantive data [4]. Although threats to marine turtles vary between species and global regions, human activities; including bycatch, marine pollution, hunting, climate change, and coastal development, exert significant pressure on populations [5]. As a result, the conservation and protection of global marine turtle populations is critical.

Rehabilitation, defined as providing care to injured or sick animals with the aim of returning them to the wild, is one technique used in the conservation management of marine turtles [6]. Although rehabilitation focuses its efforts at the patient level it can provide valuable benefits beyond the recovery and welfare of individual animals [7]. The close contact with otherwise elusive animals facilitated through wildlife rehabilitation can not only create a powerful platform for public education and awareness, but also provide a wealth of otherwise rare opportunities for data collection and scientific study [7].

Satellite tracking is one method used to study the ecology and behaviour of marine turtles which can be facilitated through rehabilitation programs [8]. To date, the majority of satellite tracking studies of marine turtles concern post-nesting migrations of adult female turtles from their natal beaches to habitual feeding grounds [8]. Such studies help to identify interconnectivity between global regions, threats along migratory corridors, and understand habitat use in the inter-nesting and post-nesting periods [9]. Although it is known that during these migrations marine turtles use a variety of environmental cues to determine their location and actively swim to stay on course, habitat use and behaviour of turtles out-with the nesting period remains less well understood [8,10].

Although less common than studies of nesting females, the few existing works into the post-release movements of rehabilitated turtles have provided useful information on the effectiveness of rehabilitation [11–13]. Released turtles, even those with significant injuries such as amputated flippers, show high survival rates in the months following release [11,13,14]. The behaviour of rehabilitated turtles has also been found to be comparable to that of uninjured animals with released animals shown to follow expected migration routes and visit known feeding grounds and nesting sites [12,15,16]. This suggests that tracking the post-release movements of rehabilitated turtles using satellite telemetry can be extrapolated to provide relevant insights into the behaviour of wild populations.

One notable gap in satellite tracking research to date is the so-called ‘lost years’ of the marine turtles’ life cycle [17]. After hatching, juvenile turtles migrate to the open ocean, where they spend the first 10–15 years of their lives [17]. Following this period some species, including green (*Chelonia mydas*) and hawksbill turtles (*Eretmochelys imbricata*), then return to neritic habitats and show high site fidelity for the remainder of their lives. Other species, including olive ridley (*Lepidochelys olivacea*) and leatherback turtles (*Dermochelys coriacea*), retain higher behavioural plasticity and remain predominantly in pelagic waters [17,18]. Very little is known about the behaviour of marine turtles in the ‘lost years’ stage of their life cycle as the pelagic lifestyle of these turtles makes locating suitable study animals challenging [19]. Although it is well understood that older juvenile sea turtles occupy similar habitats and display similar behaviours to those of adults, following the movements of smaller animals through satellite tracking has only been made possible in recent years through ongoing developments in tracking technology, particularly reductions in tag size and mass [20–22]. Although currently limited to the Atlantic basin, tracking of smaller juvenile turtles has yielded crucial insights into the behaviour and movements of juvenile turtles in the early years of life [23]. Like their adult counterparts, their overall routes are dictated by current direction, with even very small turtles actively swimming to maintain position, access resources, and follow continental shelves [19,20,22].

Another significant gap in satellite tracking research concerns olive ridley turtles: despite being the most abundant sea turtle species, olive ridley turtles are underrepresented in satellite tracking studies, accounting for just 4% of tagged turtles worldwide [24]. Although it is established that surface currents influence the post-nesting movements of the predominantly pelagic-living olive ridley turtles; research on the effects of these currents during other periods remains limited [18,25,26]. However, given the species’ vulnerability to bycatch and marine pollution whilst in the open ocean, understanding their migration routes and behaviour at other stages of their life cycle is crucial [18].

The Republic of the Maldives (Maldives) is a region of important habitat for marine turtles [27]. Both green and hawksbill turtles reside within the shallow atolls with year–round nesting of both species recorded in most atolls [27,28]. Although there is some evidence of female green turtles migrating to the nearby Chagos archipelago to nest, the degree to which Maldivian populations are connected with neighbouring regions remains unclear [29]. Olive ridley turtles are frequently spotted migrating offshore and in the inter-atoll channels, with loggerhead (*Caretta caretta*) and leatherback turtles also recorded as passing migrants [27]. A previous genetic study suggests that olive ridley turtles travelling through the Maldives originate from Sri Lankan and east Indian populations. However, the broader migration routes used by olive ridley turtles in this region remain unclear [30].

The rescue and rehabilitation of marine turtles within the Maldives is well established. Olive ridley turtles have been previously recorded as comprising the majority of rehabilitation cases in the country, predominantly through injuries caused by entanglement in ghost fishing gear [31,32]. Green and hawksbill turtles are also admitted to rehabilitation centres for a variety of reasons including illness, injuries caused by entanglement, and boat strikes [32].

Here, we use satellite telemetry to examine the behaviour of marine turtles following their release from rehabilitation centres within the Maldives. Through tracking the post-release movements of juvenile and adult olive ridley, green and hawksbill turtles we substantiate differences in behaviour between species and life stages, explore the effects of ocean currents on migration routes, assess the survival of animals in the months following their release, and infer connectivity of marine turtle populations between regions.

## Methods

The Maldives Sea Turtle Conservation Programme (MSTCP) began operating in 2011. The project aims to protect and conserve local sea turtle populations through the rehabilitation of injured and sick turtles, in addition to conducting research into their biology and ecology. This research includes satellite tracking of released individuals, monitoring of turtle nests, and photo-identification of wild turtles. The project is registered under Environmental Protection Agency (EPA) Protected Species Research Permit number EPA/2024/RC-01 and is a partnership between environmental consultancy company Reefscapers Pvt Ltd and Four Seasons Resorts Maldives. The programme has two bases: at Landaa Giraavaru in Baa atoll, and Kuda Huraa in North Male atoll. The satellite tracking research is carried out with permission from the Ministry of Fisheries and Ocean Resources (research permit no: NRP2024/78).

### Study area

The Maldives is a double chain of 26 coral atolls in the Indian ocean, lying between 07°06’N and 00°41’S and 72°32’E and 73°45’E, covering an area of over 900,000 km^2^ [33]. The tropical climate maintains a fairly constant air temperature year-round, averaging between 27 and 31 degrees Celsius, and has 2 distinct seasons; the Southwest Monsoon which runs from mid-May until the beginning of November and brings wet stormy weather from the Arabian Sea, and the Northeast Monsoon between January and March which carries dry calm weather from the Bay of Bengal [34]. These two seasons are interspersed with two inter-monsoon periods characterised by changeable weather conditions. Water temperatures vary slightly with the seasons with a high in April of 30 degrees Celsius and a low of 28 degrees Celsius in December but remain relatively constant year-round [34]. The coral reefs of the Maldives are highly biodiverse and support a wide variety of marine life, including marine turtles [35].

### Data collection and processing

All animals admitted into the MSTCP rehabilitation program had a standard set of data recorded including: species, sex (juvenile, adult male, and adult female), cause of admission into rehabilitation, details of any injuries present (e.g., presence of missing flippers, lacerations etc.), and admission date. Adult turtles were distinguished from juveniles using curved carapace length with measurements of over 60 cm in olive ridley turtles, 100 cm in green turtles, and 75 cm in hawksbill turtles being categorised as adults [36–38]. Adult male turtles were identified by the presence of a tail protruding beyond the caudal border of the carapace [39]. Cause of admission into rehabilitation was determined by a combination of clinical examination and knowledge of the animals’ circumstances, e.g., cause of admission was recorded as ‘entanglement’ in animals found caught in ghost fishing gear. At the time of tag deployment, the release date, season, animals’ release weight (in kilograms), release size (curved carapace length), and number of days spent in rehabilitation were also recorded. Release season was determined as ‘Northeast monsoon’ (January to March), ‘Southwest monsoon’ (mid-May to October), transition period 1 (April to mid-May), and transition period 2 (November to December) [35].

All animals included in the tracking study were fitted with satellite tracking tags designed for use in hard-shell turtles (‘SPOT’ and ‘SPLASH’ tags produced by ‘Wildlife Computers’) with tag model selected to ensure maximum battery life whilst remaining less than 3% of the individual’s body weight to prevent detrimental effects on swimming ability [40,41]. Tags were attached using the protocol recommended by the manufacturer [41]. All data recorded by the tags; location, time, date, data quality, as well as temperature, haul-out and depth data for some tag models, was relayed through the Argos satellite telemetry system [42].

Data was downloaded from each tag to a local server every 7 days. After a period of 2 weeks with no further data being received from a tag the date of last transmission and total number of days transmitting were recorded.

Although the Argos system provides estimates of datapoint accuracy, low-confidence locations are common, particularly over the equator where satellite passes are less frequent [43,44]. As a result, removing points based on confidence alone can often be overly rigorous [44]. Alternatively, applying algorithms to filter implausible locations effectively increases data accuracy whilst retaining valid information [43].

First, all points considered to be invalid by the Argos system, i.e., those not passing a minimum of two plausibility checks were discounted from the analysis [45]. Next, speed and azimuth filters were applied to improve the accuracy of the data with sequential points requiring travel of faster than 5 km/h or a turning angle of more acute than 20 degrees being removed from the dataset [45]. A speed threshold of 5 km/h is commonly used for hard-shelled marine turtles as, although speeds greater than this are possible, they are rarely sustained for extended periods [46]. Similarly, marine turtles do not commonly perform sharp turns whilst swimming and previous studies have considered locations leading to a turning angle of less than 20 degrees to be implausible (S1 Raw Data) [45].

Following this, the data was imported into GIS to establish the overall behaviour of each animal and calculate their initial heading, total distance travelled, average speed, and to examine the data for indications of the reason behind the tag transmission ending. Current data was extracted for each datapoint and was used to calculate swimming effort and swimming bearing at each point.

The behaviour of an animal was considered migratory if the atolls were left and not returned to, i.e., the animal travelled out-with the Maldives. The average number of days taken for an animal to migrate was calculated by subtracting the difference between the date the animal was released and the date it was recorded leaving the atolls. A Pearson’s correlation was used to test whether the length of time an animal spent in rehabilitation affected how quickly they left the atolls and began migrating (for animals which migrated).

The initial heading of an animal was the bearing of the line of best fit between satellite locations recorded in the first 10 days after migrating animals left the atolls and their protection from prevailing currents.

Total distance travelled was calculated as the distance in kilometres between consecutive points from a single animal with average speed taken as the total distance travelled divided by the number of days the tag transmitted for.

**Fig 1:**
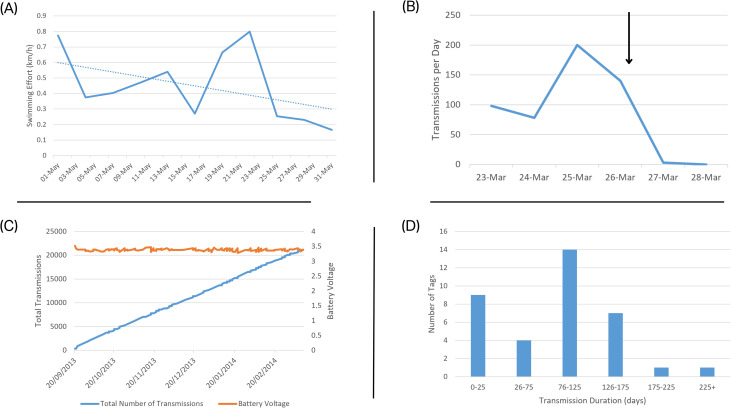
Examples of data used to determine cause for the end of transmissions. **(A)** Decline in swimming during the final month of transmission for one animal, with a pronounced reduction occurring in the final week suggesting mortality. **(B)** Number of daily transmissions, showing a sharp decline and cessation after release (release date indicated by arrow), consistent with suspected tag malpositioning. **(C)** Example of temporal trends in total transmission count and battery voltage, used as indicators of battery depletion. **(D)** Duration of tag transmission after release, ranging from 1 to 349 days across the study.

To determine the likely causes of transmission cessation, data from each satellite tag was inspected for common indicators of tag failure. These included: battery failure (characterized by battery amplitude dropping below 3 amps, steeply declining over the final few transmissions, or the total number of transmissions falling within the expected lower range for battery exhaustion); tag detachment (indicated by an abrupt cessation of transmissions without signs indicating an alternative form of tag failure, or a sudden increase in transmission frequency; suggesting the tag had surfaced, combined with a loss of active swimming); animal mortality (evidenced by a loss of active movement, absence of diving behaviour, and often an erratic or reduced frequency in transmission rate); tag malfunction (identified through abnormal readings outside expected biological or operational parameters); and biofouling (inferred from a gradual decrease in transmission rate over time) (Fig 1) [47,48]. Although GPS data was available for all tracked animals, historical data loss of telemetry records meant that diagnostic data was unavailable for some tags. As a result, full assessments of the cause of transmission cessation, including evaluation of battery status and tag performance, was not possible for a subset of deployments.

Current data was extracted using the coordinates and time for each data point from the Ocean Surface Current Analysis Realtime (OSCAR) Sea Surface Velocity 1/3 degree resolution global 5-day composite dataset. This dataset was the only dataset spanning the full study duration and uses sea surface height, surface vector wind, and sea surface temperature data gathered from satellites and in-situ instruments to directly estimate near-surface horizontal current velocity.

Swimming effort was considered to be the degree of animal displacement not accounted for after considering current angle and velocity. The difference between current and swimming angle was used to determine the extent that animals were swimming with or against currents.

T-tests were used to examine variations in tracking duration and average speed between species whilst linear models determined the effects of bodyweight and the loss of flippers on overall speed. Linear models were also used to test the impacts of bodyweight, ocean depth, and current strength on swimming effort. Finally, Fisher’s test for count data was used to test for differences in initial heading during the different seasons.

The extent to which animals swam with or against the prevailing current was estimated by subtracting the bearing of the current from the direction of swimming effort at a given point in space and time to give an angle of difference between 0 and 180 degrees. The frequency of occurrence of the resulting angles was compared using the Rayleigh statistic of uniformity both across the whole dataset and for each species. The impact of bodyweight on this angle was tested using a linear model. Bathymetry data included in this model was extracted for each location from the General Bathymetric Chart of the Oceans (GEBCO) dataset [49].

## Results

### Animal overview

The post-release movements of thirty-five rehabilitated sea turtles (olive ridley (n = 20), green (n = 12), and hawksbill (n = 3)) released by the MSTCP were tracked between March 2012 and June 2024. Animals were released after meeting several pre-requirements including: the ability to swim strongly, presence of normal diving ability and feeding behaviours such as foraging or hunting, maintaining or gaining bodyweight, being off all medications for a minimum of 14 days, any injuries being in the maturation phase of healing or later, and the absence of disease. Whilst the tagged olive ridley turtles were admitted to rehabilitation for entanglement injuries (n = 17) or buoyancy syndrome (n = 3), the green and hawksbill turtles were mostly released from the centres as part of a since-discontinued headstart program (n = 15). This group also included several young juveniles surrendered by locals and those confiscated by the government after being found kept as pets (S2 Summary and overview of tracking data).

Of the twenty olive ridley turtles, 11 were juveniles, 7 adult females, and 2 adult males, which spent an average of 210 days in rehabilitation (range: 3–1741 days) and had a mean bodyweight of 22.3 kg (range = 7–33.9). Six of these animals were missing flippers as a result of their injuries. Conversely, the green and hawksbill turtle juveniles were released at approximately one year of age and had a mean bodyweight of 4.1 kg (range = 3.3–4.7 kg) and 3.7 kg (range = 2.7–4.7 kg) respectively. For this reason, species and bodyweight were considered to be non-independent for the purposes of data analysis.

### Tracking duration

The average tracking duration across the project was 92 days (range = 1−349 days, median = 93 days, n = 35) with total distances travelled varying between 27.4 km and 4959.2 km (mean = 2345.4 km, n = 25). However, duration of tracking period varied significantly between species with tags remaining active for significantly fewer days in hawksbill turtles (mean tracking duration = 12.7 days, range = 4−25 days, n = 3) than for green (mean = 102 days range = 3−349 days, n = 12, t(12.1)=−3.31, p = 0.006) or olive ridley (mean = 98 days, range = 1−220 days, n = 20, t(19.4)=−5.68, p=<0.001) turtles. There was no significant difference in tracking duration between green and olive ridley turtles (t(17.0) =−0.14, p = 0.89).

### End of tracking period

Several potential causes for transmission cessation were identified from both tag performance and movement data (Fig 1). Two tags were suspected to have been malpositioned on attachment, as they transmitted normally prior to release in the rehabilitation centre whilst the turtles were held in shallow pools, but ceased transmitting within a day after release, suggesting these tags did not break the surface with normal swimming behaviour (Fig 1B). Of the tags where tag status information was available (n = 13) three showed abnormal or biologically implausible sensor readings indicating tag malfunction. One tag, for example, began recording erroneous depth readings (between -3000m and -4000m) shortly before the tag stopped transmitting. One animal was a possible mortality event, with mean swimming speed declining from an average of 0.54 km/h to 0.21 km/h during the final week of transmission (Fig 1A). Two tags were considered possible cases of antenna malfunction or premature detachment, as transmissions initially occurred normally before becoming erratic and ceasing between a few days to just over one week after. As both these tags transmitted for less than one month it was considered unlikely that this pattern was caused by biofouling. Seven tags with longer transmission periods were thought to be affected by biofouling: showing progressively sparse and irregular transmissions during the weeks prior to transmission cessation. None of the tags for which status data was available showed indicators of battery exhaustion (Fig 1C).

**Fig 2:**
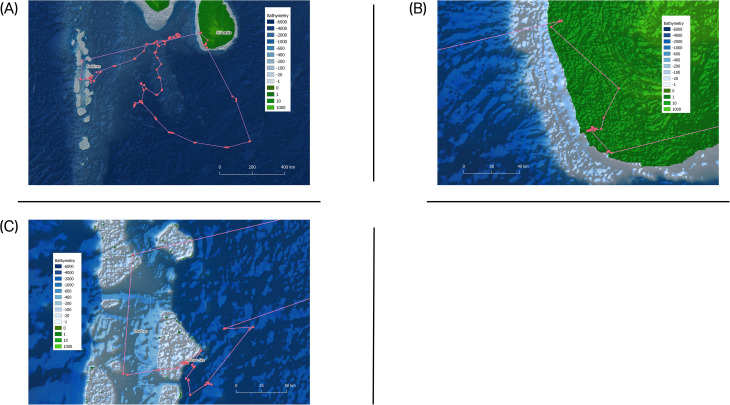
The tracking period of one juvenile green turtle showing apparent bycatch and possible re-release. **(A)** overview of the tags journey. **(B)** The tag transmitted from the island of Sri Lanka for 72 days. **(C)** After this, transmissions were received intermittently for a further 10 weeks from close to the original release site. Figure produced by the authors in QGIS using data derived from the GEBCO 2025 Grid [49].

One tag was inferred to be a likely fisheries interaction (Fig 2). Following a period of rapid movement, the tag transmitted from the island of Sri Lanka for 72 days, first near the coastal fishing town of Mirissa and then from the capital of Colombo (Fig 2B). Twelve days after this the tag began transmitting again from within the Maldives, approximately 775 km away, where it remained until finally stopping transmitting 10 weeks later (Fig 2C).

In other tags with an absence of diagnostic indicators suggesting alternative explanations, tag detachment was considered a possible reason for transmission cessation.

### Behaviour and movements

**Fig 3:**
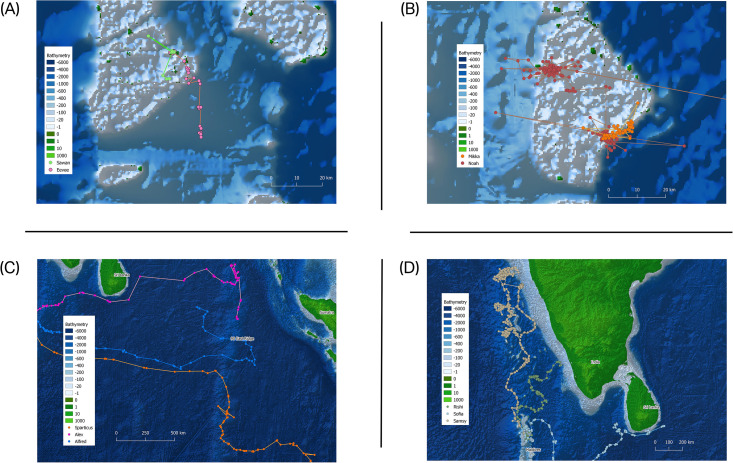
Overview of the behaviour patterns displayed by turtles after release. **(A)** Short tracking durations in several animals did not allow accurate classification of behaviour. **(B)** Several green and hawksbill turtles remained resident within the Maldives after their release. **(C)** Animals which migrated travelled significant distances often used oceanographic features such as deep-sea ridges and continental shelves **(D)** to navigate. Figure produced by the authors in QGIS using data derived from the GEBCO 2025 Grid [49].

Of the 35 tracked turtles, 25 animals migrated beyond the atoll they were released from: travelling into open ocean. Of the remaining 10 animals which did not migrate, 6 of these had a very short tracking duration (less than 6 days; the meantime taken for animals to leave the atolls) where it was not possible to accurately determine their behaviour (Fig 3A). All of the olive ridley turtles tracked for a period of longer than 6 days (n = 16) migrated along with 81% of the green turtles (n = 11) and 0% of the hawksbills (n = 2).

All nine of the adult olive ridley turtles migrated (average tracking duration 120 days) with none returning to the release site.

For animals which migrated, the mean duration between release and leaving the atolls was 6 days (range = 1−23 days, mode = 3 days). There was a slight positive correlation between the length of time an animal spent in rehabilitation and the number of days taken to begin migrating (r = 0.33, CI = −0.095, 0.653). However, this correlation was not statistically significant (p = 0.124).

Of the animals which did not leave the Maldives, one juvenile green turtle was recorded residing within the atoll it had been released from for almost a year (349 days) (Fig 3B). This animal had been captive reared for almost 15 months (446 days) and on release weighed 4.2 kg with a straight carapace length of 32 cm.

Interestingly, several of the animals which migrated were noted to follow significant geographic features including continental shelves and deep ocean ridges (Fig 3C, 3D).

### Speed of travel

The average speed of travel across the study was 23.85 km/day (range = 2.65–50.45 km/day, n = 26). Although migrating animals were recorded travelling at greater speeds (mean = 25.79 km/day, range = 8.33–50.45 km/day, n = 25) than those which remained resident (mean = 7.97 km/day, range = 2.65–13.3 km/day, n = 4) this finding was not statistically significant. Additionally, there was no significant effect of species (t(20.1)=0.58, p = 0.56), bodyweight (F = 0.01, p = 0.92), or of loss of flippers (F = 0.34, p = 0.72) on speed of travel.

### Initial heading

**Fig 4:**
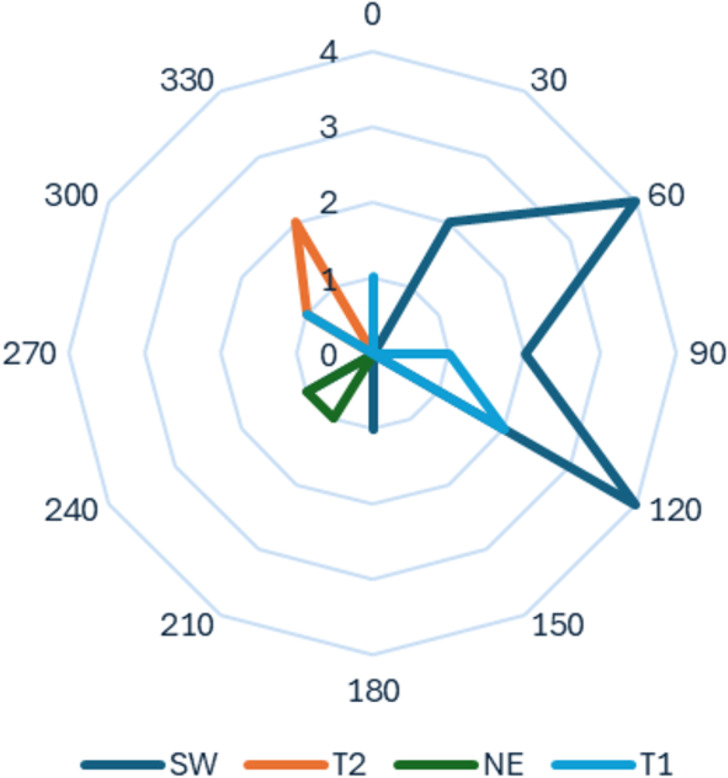
Radar plot showing the count of initial heading (degrees) of migrating turtles departing Maldives during different seasons.

Of the 25 turtles which migrated during their tracking period, 2 animals were released during the northeast monsoon, 15 during the southwest monsoon, 5 during the first transition period, and 3 during the second transition period. The initial heading of these animals after leaving the atolls of the Maldives was found to vary significantly between these periods (p = 0.013) with animals heading correlating strongly with the direction of the prevailing current (Fig 4).

### Swimming angle and current

The relationship between swimming angle and current bearing for animals which migrated was not uniformly distributed and instead showed moderate clustering around a mean direction (Z = 0.6, p = 0, mean = 74.1 degrees, median = 65.2 degrees). This was also true for both olive ridley (Z = 0.62, p = 0) and green (Z = 0.65, p = 0) turtles independently, although with a slightly stronger clustering effect in green turtles, showing that both species swam actively with and against ocean currents, but showed a higher tendency to move in the same direction as the water movement.

**Fig 5:**
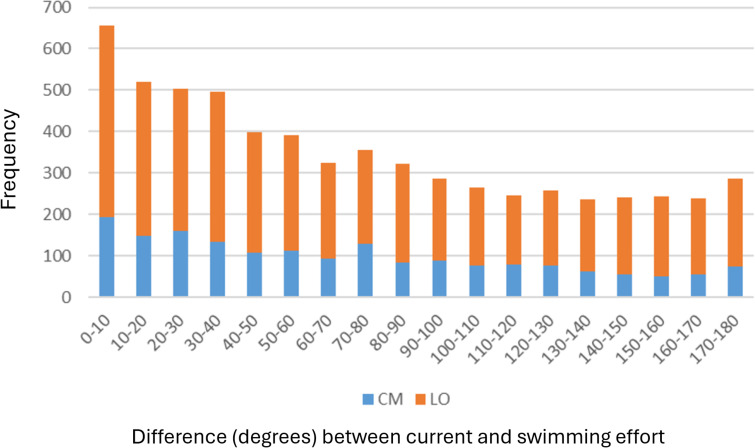
Count of angle of difference (degrees) between swimming bearing and current bearing in migrating olive ridley (orange/LO) and green (blue/CM) turtles.

Bodyweight also had a slight positive effect on the angle of difference between current bearing and swimming angle with heavier animals swimming 0.19 degrees further from the current per kilogram of body mass (F = 9.068 p = 0.003) (Fig 5).

### Factors affecting swimming effort

Whilst current strength was found to have a positive effect on swimming effort (𝛽 = 0.856, SE = 0.028, p=<0.0001), both bodyweight (𝛽 = −0.003, SE = 0.0004, 0=<0.0001) and ocean depth (𝛽 = −0.00006, SE=<0.0001, p=<0.0001) had a negative effect. This indicates that animals with higher body mass had increased swimming effort compared with lighter animals and that swimming effort was higher both in stronger currents and shallower water.

## Discussion

As the first satellite tracking study of marine turtles in the Maldives, this research provides valuable insights into the post-release movements and behaviour of rehabilitated individuals in the Indian Ocean. Our findings demonstrate that species and life-stage specific behaviours broadly align with expectations from wild populations in other global regions, supporting the validity of rehabilitation-based tracking as a tool for ecological study. In particular, the inclusion of under-represented groups such as olive ridleys and juvenile turtles extends current knowledge and helps to address long-standing gaps in the satellite tracking literature. The high survival and migration capacity observed, even in animals released after significant injuries or prolonged rehabilitation, underscores both the resilience of marine turtles and the broader conservation value of rehabilitation programmes. Although the reasons for transmission cessation differed somewhat from those reported in previous literature, the broader movement patterns observed were largely consistent with previous findings [48]. In concurrence with comparable tracking studies it was found that, although ocean currents play a strong role in influencing the movements of turtles in open ocean, both juvenile and adult turtles swim actively within currents and use oceanographic structures and features such as continental shelves to navigate ocean basins [20,50]. This study highlights the interconnectivity of ocean basins for marine migrants and has far-reaching implications for both the conservation management of this group both within the Indian Ocean and globally.

### Species and life-stage differences

Firstly, clear differences between species were evident, both in the reasons turtles were admitted to rehabilitation and in their behaviour after release. Species-specific causes of injury and admission have been previously documented in the Maldives: injuries to olive ridley turtles of varying ages are most common following entanglement in ghost fishing gear, whilst green and hawksbill turtles hatchlings are vulnerable to captive-rearing in the pet trade [30–32]. The cases included in this study reflect these patterns, with juvenile and adult olive ridleys mostly admitted due to entanglement injuries and the majority of green and hawksbill cases being captive-raised juveniles.

Post-release movements also reflected expected species and life-stage differences. All tracked olive ridleys migrated away from their release sites, consistent with their pelagic and migratory lifestyle [51,52]. This behaviour is well documented in olive ridley turtles in this region with studies from Sri Lanka, eastern India, and other regions recording long-distance dispersal [52,53]. Although the behaviour of adult sea turtles can vary between sexes, particularly during breeding periods when males may migrate shorter distances and more frequently than females, both male and female olive ridleys in the present study migrated away from their release sites and travelled comparable distances [54]. Whilst several animals made significant changes in heading during their tracking periods with some travelling back towards the Maldives archipelago, these changes coincided with seasonal shift in currents. As the Maldives are not a significant breeding area for olive ridley turtles, nor noted as a country where they commonly reside, these movements were unlikely to be the result of mating driven behaviour with the Maldives as an end goal [27].

In contrast to the migratory behaviour of olive ridley turtles, juvenile green turtles displayed a mix of migratory and resident behaviour, consistent with their shift from pelagic to neritic habitats at as little as a few years old, with some individuals remaining resident for extended periods, including one tracked for nearly a year [55]. Although the three juvenile hawksbill turtles included in this study did not appear to migrate, the small sample size and short tracking durations limited the conclusions that can be drawn for this species. Similar variability in behaviour has been recorded in previous studies tracking the behaviour of immature green and hawksbill turtles. One study of 23 wild-caught juvenile green and hawksbill turtles found that although a few individuals migrated hundreds of kilometres, most individuals remained in the vicinity of the tagging site [56]. A second study of 6 juvenile green turtles found that, whilst wild-caught animals remained close to their release site, captive reared individuals travelled significant distances, similar to the pattern observed here [57]. These apparent differences between captive-raised and wild-caught individuals suggest that post-release movements of immature turtles are influenced by both ontogenetic stage and prior habitat use. Wild-caught juveniles are likely to have recruited to neritic developmental habitats prior to tagging, whereas captive-reared turtles may be released before developmentally reaching residency-stage behaviours.

Overall, these findings suggest that life history and species-specific traits strongly shape post-release behaviour of all three species studied, with natural instincts overriding multiple years in rehabilitation, even for animals reared in a captive environment. This confirms that rehabilitated turtles are a reliable proxy for the behaviour of wild individuals and should be considered a valid source of data for future ecological and conservation studies.

### Rehabilitation outcomes and survival

In addition to providing valid data on wild turtle behaviour, the tracking durations and movements observed here demonstrate that turtles can successfully re-integrate into the wild following rehabilitation. Turtles were found to actively swim after their release, indicating, with one possible exception, their ability to survive and thrive. Consistent with previous studies, even significant injuries such as flipper amputations did not affect swimming performance, proving rehabilitation and reintroduction to be successful in these cases [11]. Although turtles with severe long-term injuries may be unable to contribute substantially to population recovery—for instance, individuals with missing flippers may have a reduced success in mating and nesting—their survival following entanglement underscores the value of rehabilitation both for individual welfare and conservation outcomes [7,58].

### Causes for the end of transmissions

Previous satellite telemetry studies of marine turtles have identified battery exhaustion as the most prevalent cause of transmission cessation [47,48]. In contrast, battery life did not appear to be a major limiting factor in the present study. Instead, although several tags show indications of biofouling affecting performance, many ceased transmitting abruptly with few signs to indicate the cause, thought to be possibly the result of premature detachment. It is likely that, with tracking durations notably shorter than recent comparable studies (median 92 days here, compared with the 251 days noted by Hays et al., 2021), transmissions ceased for alternative reasons before reaching the limits of their batteries. However, as battery exhaustion could not be assessed for all individuals, its contribution to transmission cessation in this study is likely underestimated.

There are several possible explanations for the comparatively short tracking durations observed in this study. Firstly, hawksbill turtles exhibited significantly shorter tracking durations than other species, reducing the overall mean tracking duration. It is possible that the irregular and smooth carapace morphology of this species reduced the effectiveness of tag adhesion, whilst the species’ propensity for resting wedged beneath rocky outcrops may have increased the likelihood of tag detachment or antenna damage [59]. Several other tags also ceased transmitting shortly after turtles travelled to reef-associated habitats, further suggesting that contact with reef substrate may have contributed to transmission failure or tag detachment (S2 Summary and overview of tracking data).

Attachment methodology may also have contributed to reduced tag retention or transmission performance. Regular staff rotations within the long-term project meant that, despite following established written protocols, many personnel were conducting tag attachments for the first time. This is supported by at least two probable cases of tag malpositioning, evidenced by tags transmitting normally prior to release whilst the animals were held in shallow pools, but ceasing shortly afterwards once turtles resumed natural swimming behaviour.

Lastly, as a relatively high proportion of tracked individuals were juveniles, it is possible that ongoing shell growth contributed to the loosening and premature detachment of some tags [48]. However, as most tags only transmitted for a few months it is unclear how much of a role this may have played.

One case was of particular note: following a period of rapid directed movement, one tag began transmitting from land near the coastal fishing town of Mirissa in Sri Lanka, strongly indicating bycatch and subsequent transport by humans. Unusually, the tag later resumed transmissions from within the atolls of the Maldives. As the time elapsed between these locations would have permitted physiologically viable swimming speeds, particularly with assistance from prevailing currents, one unlikely explanation is that the turtle was incidentally captured, brought ashore, and subsequently released, ultimately returning to within several hundred metres of its original release location. However, this narrative is impossible to prove with telemetry data alone.

### Seasonal and oceanographic drivers of behaviour

Despite the ambiguity surrounding the interpretation of some individual tracking outcomes, the ability of most rehabilitated turtles to survive and thrive after release was evident, and their movements remained closely tied to seasonal oceanographic patterns. Initial headings of migrating animals after release closely followed the prevailing monsoon currents and confirm that large scale ocean currents play a major role in shaping the dispersal and migrations of marine turtles in this region. However, as noted in previous studies, turtles were not passive drifters and actively swam both with and against prevailing currents, with a greater tendency to travel in the same direction as the water flow [20,50,60].

Bodyweight was one factor which played an important role in turtles’ ability to swim in ocean currents. Animals with greater body mass showed higher uniformity in the angles they swam at in relation to the prevailing current than smaller, lighter animals; suggesting that bigger animals were able to swim more powerfully through currents. Although there were apparent differences between species with olive ridley turtles showing a higher uniformity of swimming directions compared with green turtles, this is likely compounded by the differences in bodyweight between the two groups.

In addition to influencing swimming direction within currents, body weight also affected swimming effort with larger animals exhibiting a larger magnitude of output. Oceanographic conditions also influenced swimming effort, with animals showing increased locomotor output in stronger currents and in shallower water. These additional factors indicate animals’ active management of their position to exploit resource rich habitats or to navigate along key habitat features like continental shelves and ridges. The ability to actively swim with or against prevailing currents allows turtles to navigate effectively across large distances, maintain access to foraging areas, and potentially reach neighbouring regions [61,62].

This evidence was further supported by overlaying turtle routes onto oceanographic features, which revealed that several individuals showed consistent associations with continental shelves and oceanic ridges such as the 90°E Ridge and the continental shelf areas around Sri Lanka (Fig 3C, 3D). Upwellings created by oceanographic features have been shown to provide foraging opportunities for a variety of marine megafauna with migration times and routes of animals often coinciding with productive upwelling zones [61,62]. It is likely that the rehabilitated turtles studied here exploit these same resources and actively maintain their position in these productive zones to maximise foraging opportunities.

Together, these results demonstrate the strong influence of environmental conditions and oceanographic features on post-release movements and highlights the adaptive behaviour of turtles in response to environmental cues. Understanding the relationships between animal behaviour and oceanography is crucial for conservation planning, for example for identifying migration corridors, assessing population connectivity, and designing measures to mitigate threats. Given that migration routes of olive ridley turtles in the Indian Ocean are still poorly understood, the 90°E Ridge could represent a key feature influencing their movements and merits further investigation. Further work to integrate telemetry with fine-scale current data would better quantify swimming effort and allow in-depth evaluation of the influence of currents on animal movements and behaviour in this area.

### Regional connectivity

The migratory tracks of both olive ridley and green turtles show a wide range of dispersal patterns from the Maldives with individuals travelling to the waters surrounding Sri Lanka, along both the east and west coast of India, Lakshadweep, the Arabian Sea, the west Indian Ocean, the Andaman Islands, and as far as the Cocos (Keeling) Islands.

This diversity of locations has particular implications for the regional connectivity of green turtle populations. In the Maldives green turtles have been found to nest year-round with no fixed mating season [27,28]. With hatchling emergence occurring across all months of the year, dispersal direction will vary significantly depending on the season and prevailing currents. In this way, the Maldives may serve as a source population, contributing juveniles to multiple regions across the Indian Ocean. This is supported by a previous study from Chagos Archipelago, located approximately 600 km south of the Maldives, where an adult female turtle was tracked returning to foraging grounds in the Maldives [29]. Although no such connection with Chagos was observed among the individuals tracked in this study, this is likely due to the small sample size rather than the absence of a true link.

In contrast to green turtles, previous genetic studies of olive ridley turtles in the Maldives suggests that the majority of animals found entangled in ghost nets originate primarily from Sri Lanka and the east coast of India [30]. Populations from these regions have also been shown to be relatively genetically distinct, with limited mixing with neighbouring groups [53]. Given this restricted population connectivity, timing the releases of rehabilitated individuals may be particularly important for maximizing the probability of turtles returning to their source regions. Releasing individuals during seasons when currents flow eastwards towards Sri Lanka could therefore enhance the likelihood of successful reintegration into natal populations, whilst releases during westward-flowing monsoon periods may instead transport them away from their primary ranges and into remote oceanic regions. Further work to integrate satellite telemetry with particle trajectory modelling would provide a more comprehensive understanding of the dispersal mechanisms underlying movements in both species.

## Implications and conclusions

This study provides rare insights into the movements and behaviour of rehabilitated sea turtles in the Indian Ocean, contributing valuable information for regional conservation planning and support the long-term management of these ecologically important species. Here, we show that rehabilitated turtles can survive, migrate, and behave comparably to wild individuals, even following significant injuries. This demonstrates that the rehabilitation and reintroduction of marine turtles is justified for the welfare of individuals, and that such animals are a valid proxy for studying the movements and behaviour of wild individuals after their release.

Ocean currents were shown to exert a strong influence on turtle movements, driving dispersal into multiple ocean basins. Tracking juvenile turtles in this way also provided valuable data on the poorly understood “lost years”, helping to refine models of dispersal, survival, and habitat use during these less-understood early life stages. Both adults and juveniles actively swam within ocean currents and adjusted their routes to maintain position or navigate key features such as continental shelves and oceanic ridges.

These extensive migrations highlight the interconnectivity of ocean basins and re-iterate the need for international cooperation in managing and protecting vulnerable species. Despite their resilience, marine turtles remain vulnerable to a plethora of threats: conservation efforts should focus on olive ridley turtles, which are frequently affected by ghost gear entanglement and remain under-represented in global tracking datasets. Overall, this study highlights both the strengths and vulnerabilities of these species, as well as several key knowledge gaps, emphasizing the need for further study into the behaviour of marine turtles in open ocean to inform targeted conservation strategies.

## Supporting information

S1 Table Raw Data. (CSV)

S2 File Summary and overview of tracking data. Figures produced by the authors in QGIS using data derived from the GEBCO 2025 Grid [49].
(DOCX)

S3 Fig Striking image. Figure produced by the authors in QGIS using data derived from the GEBCO 2025 Grid [49].
(JPEG)
