# Supplementary material for: Tracking rehabilitated sea turtles in the Indian Ocean using satellite telemetry: Insights into behaviour, ecology, and conservation implications
Source: PLoS One. 2026 Jun 26;21(6):e0351541. doi: 10.1371/journal.pone.0351541 (PMC13308851; doi:10.1371/journal.pone.0351541)

Summary of patient information for animals included in the study


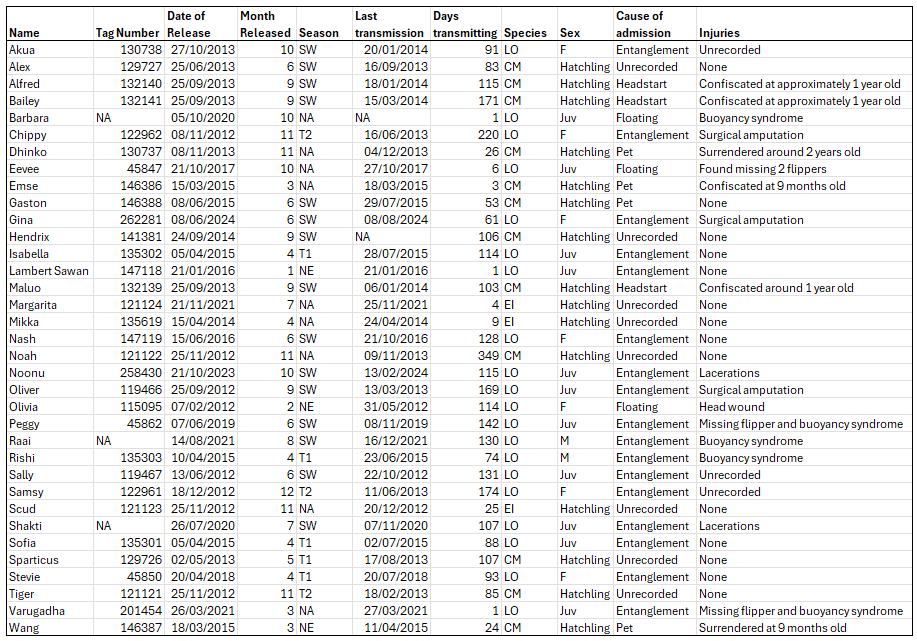


cont..
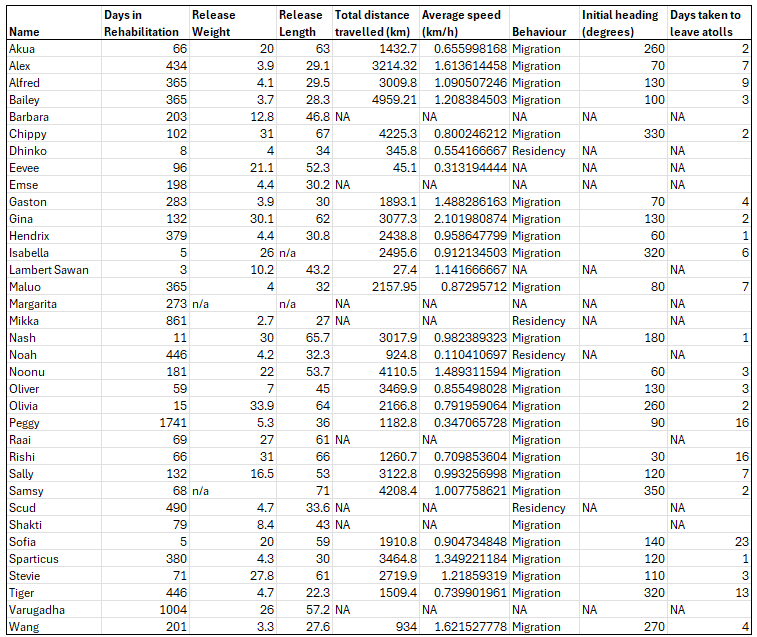


Images showing the routes taken by individual turtles

Akua


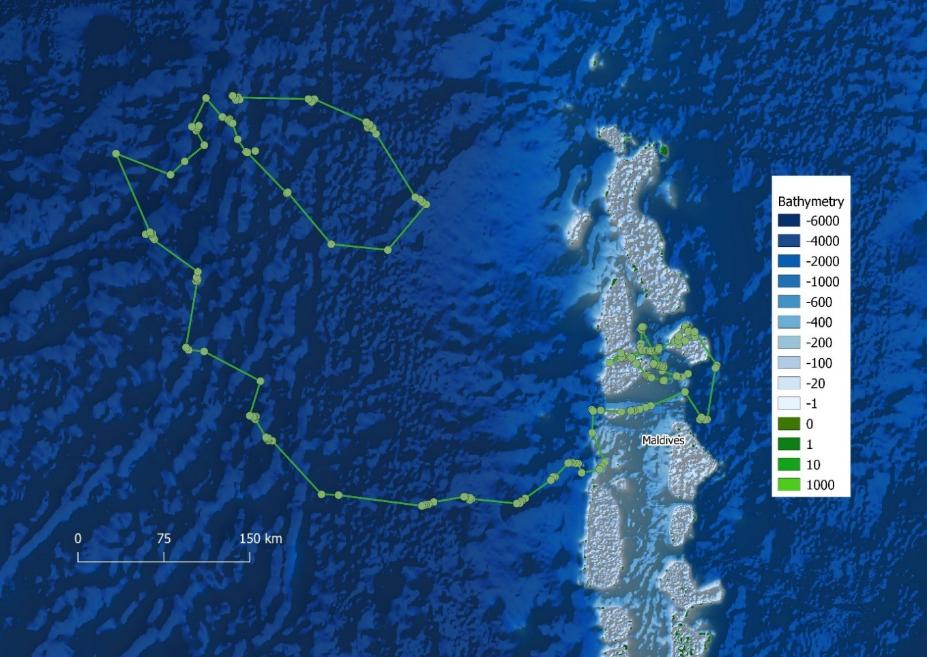


Alex
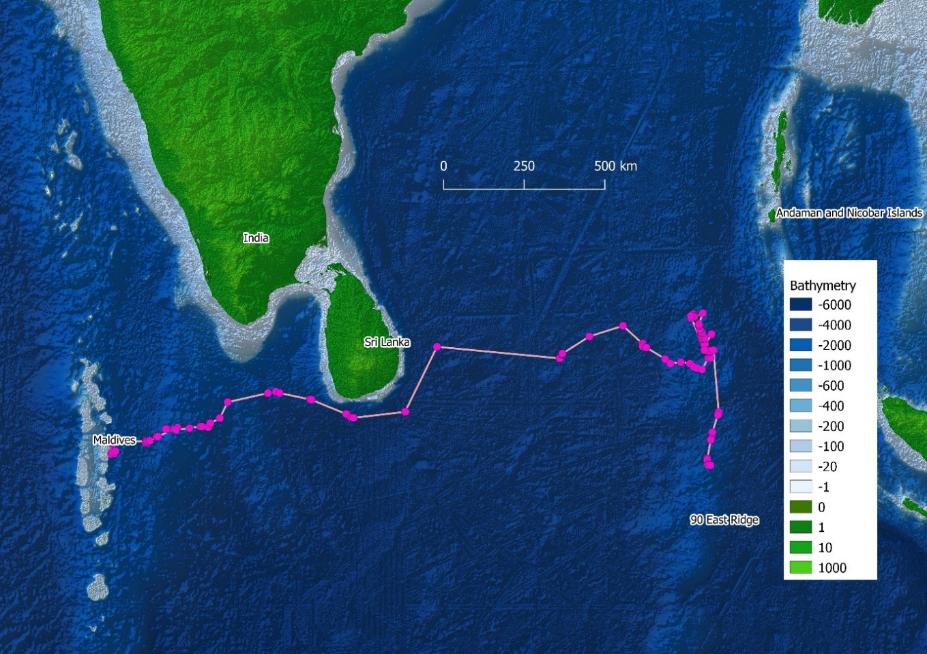


Alfred
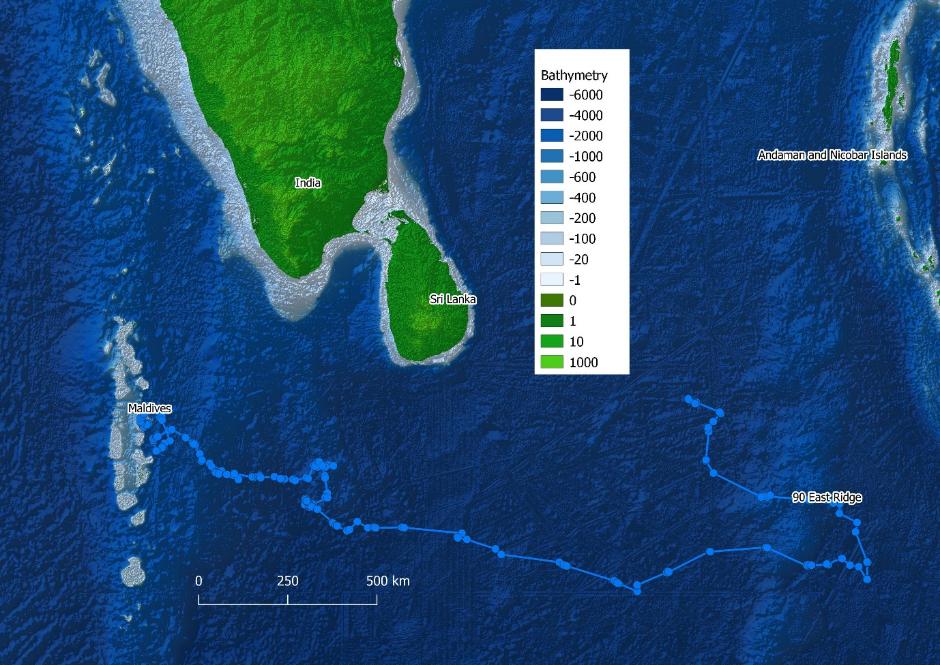


Bailey
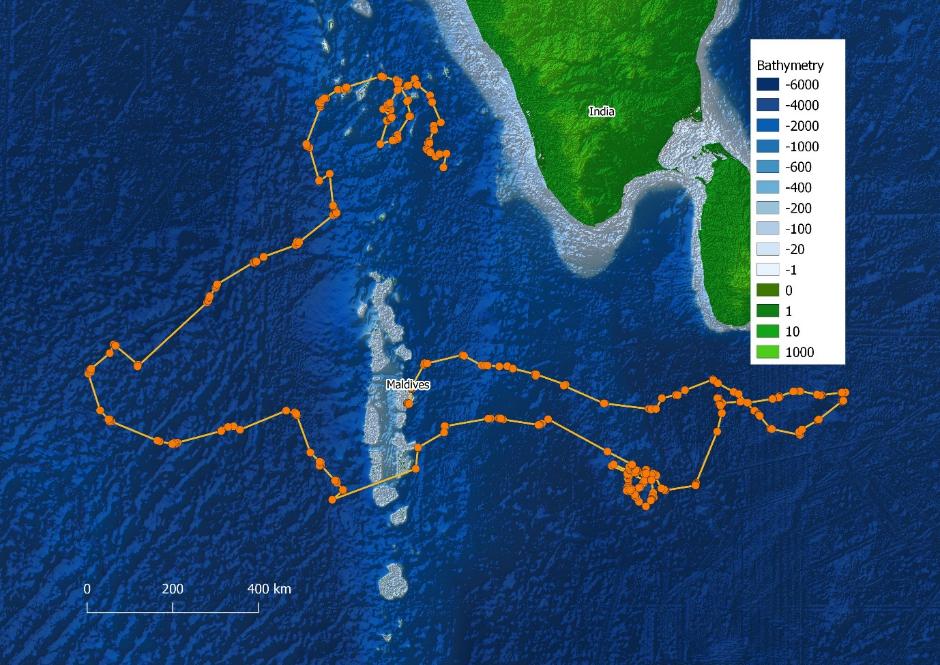


Chippy
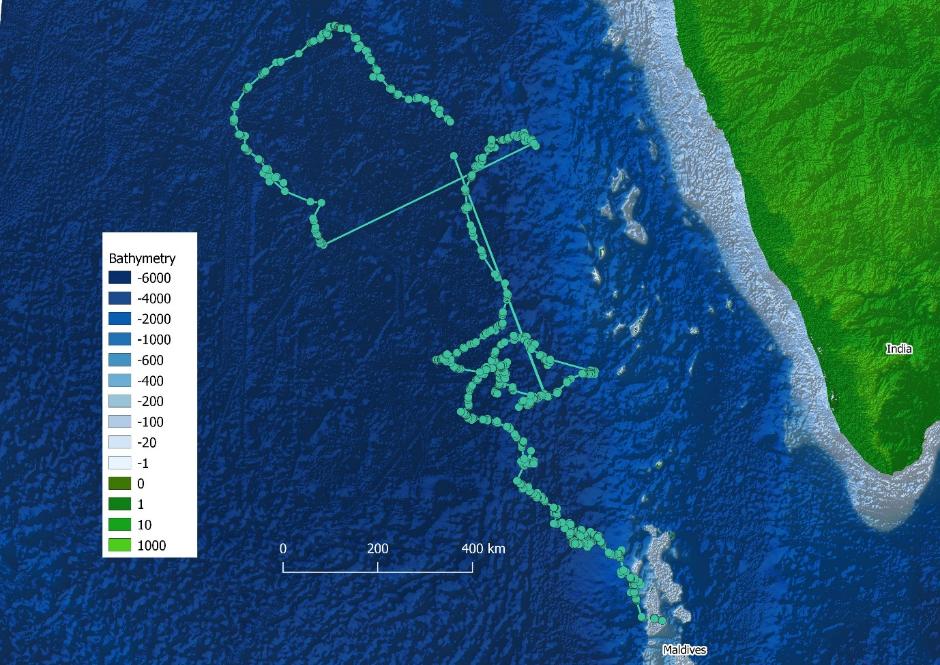


Dhinko
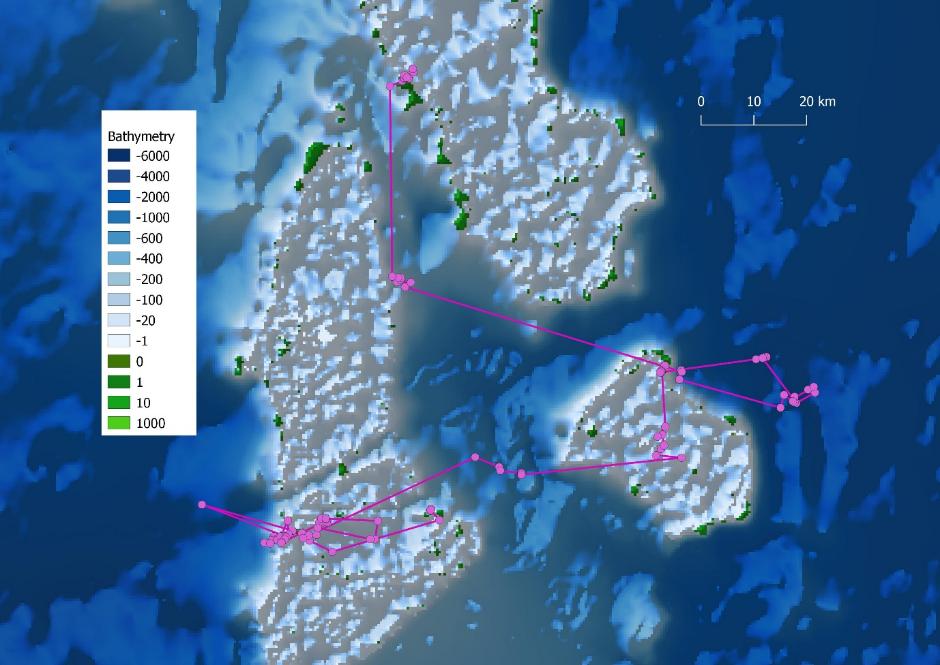


Eevee
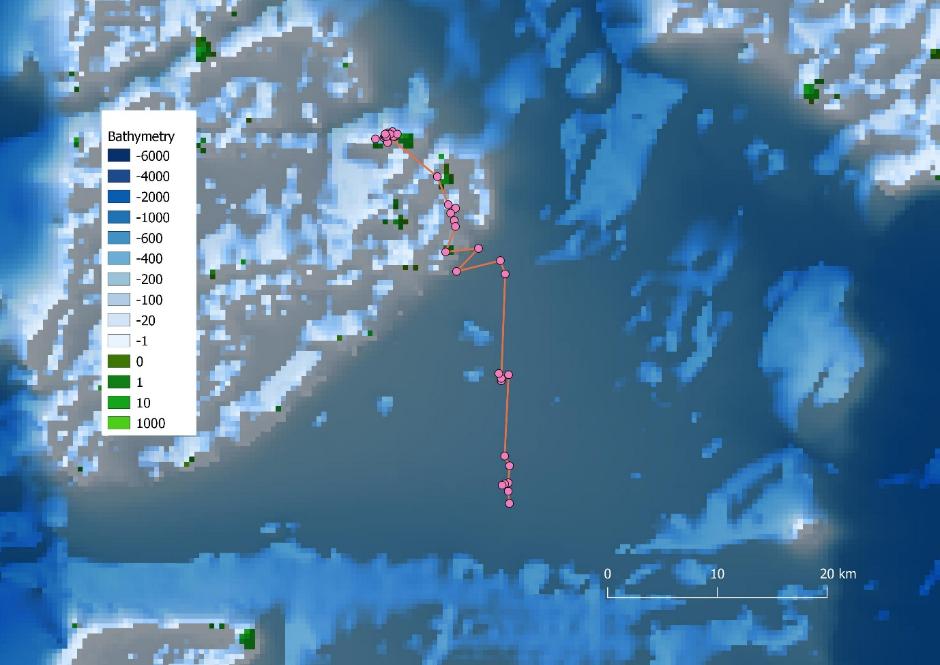


Esme
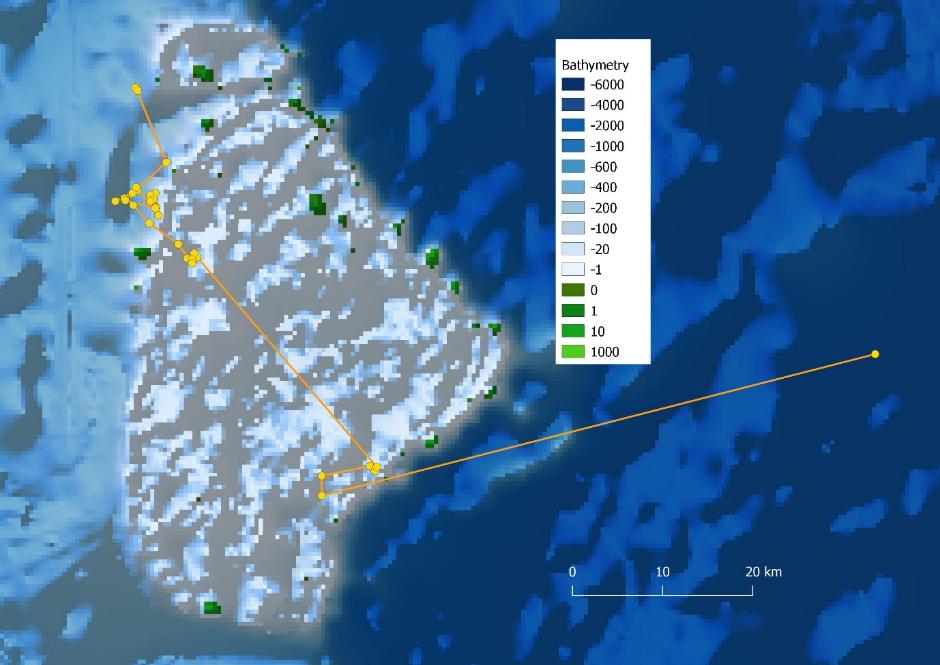


Gaston
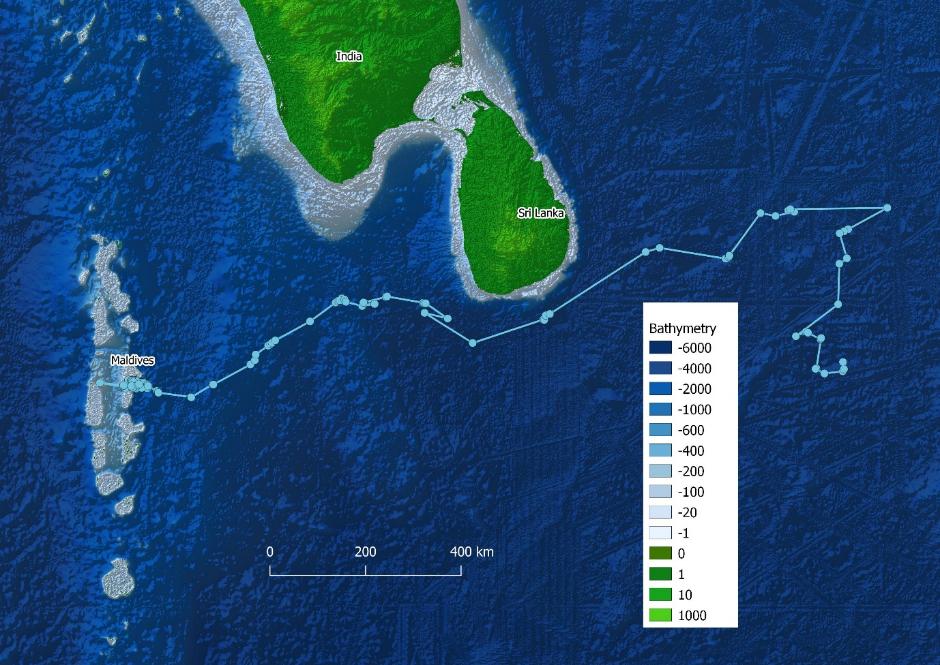


Gina
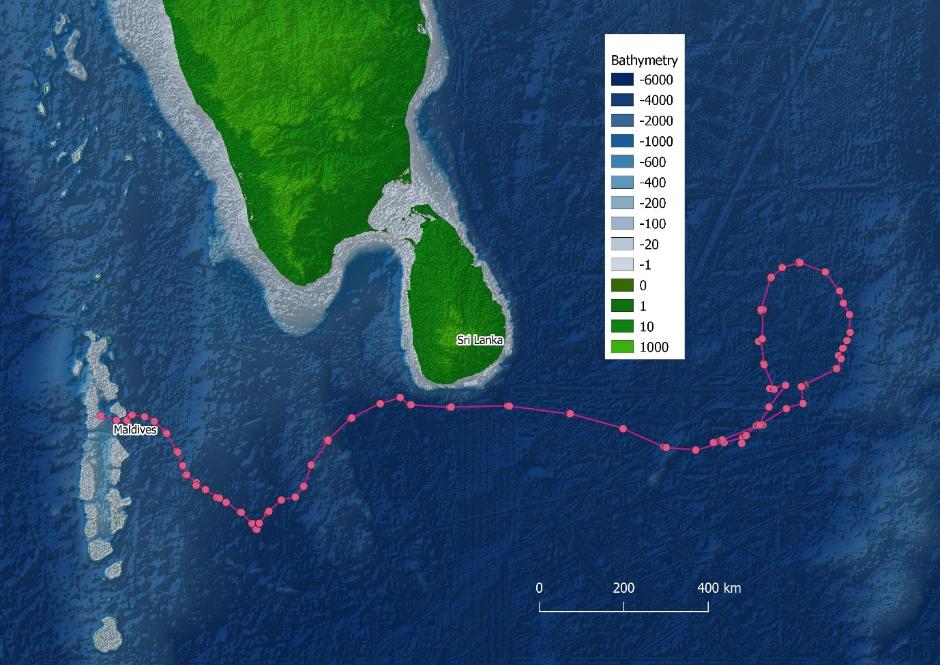


Hendrix
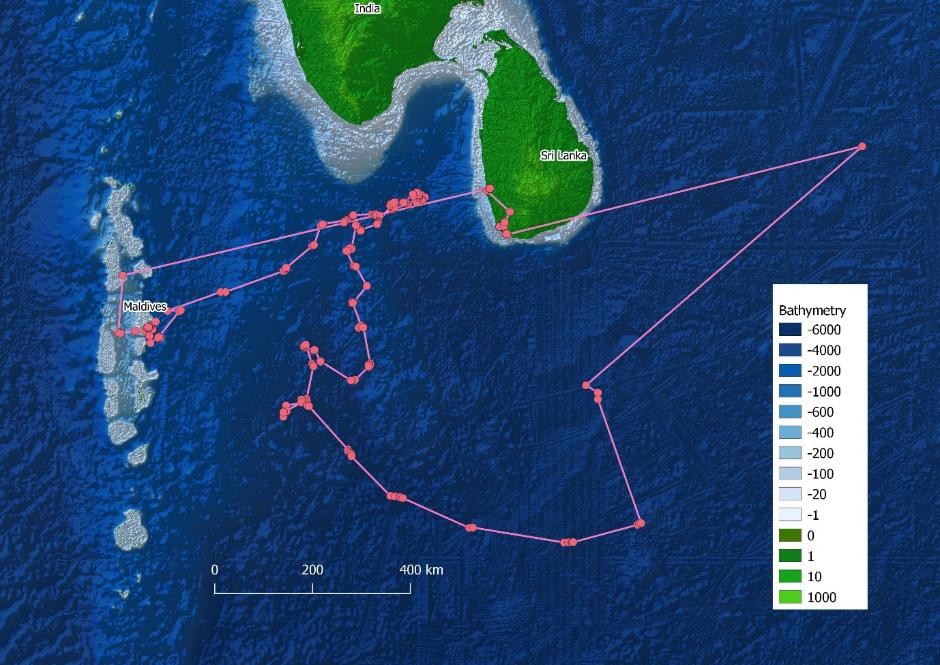


Isabella
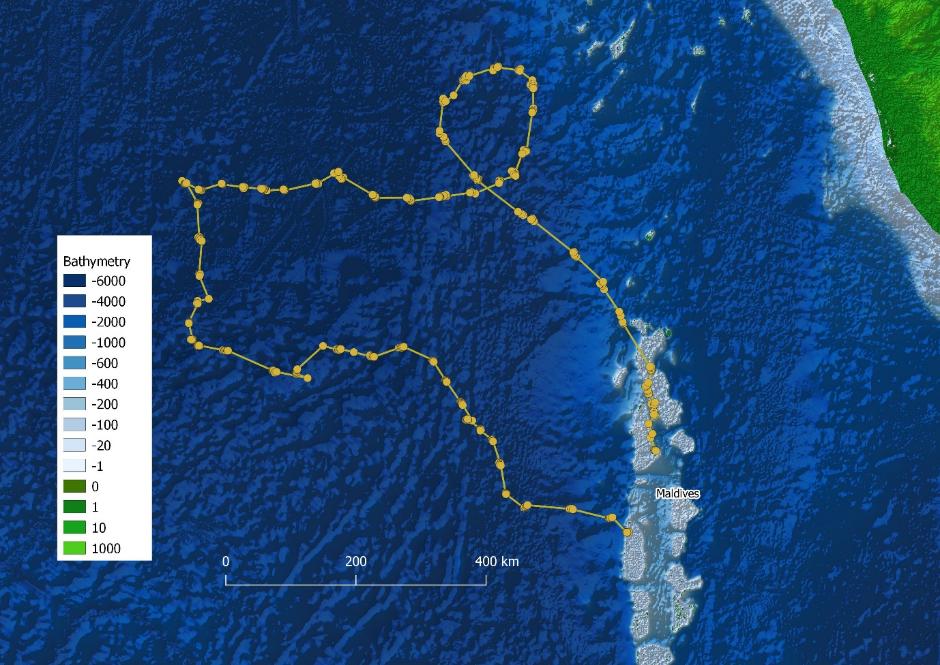


Malou
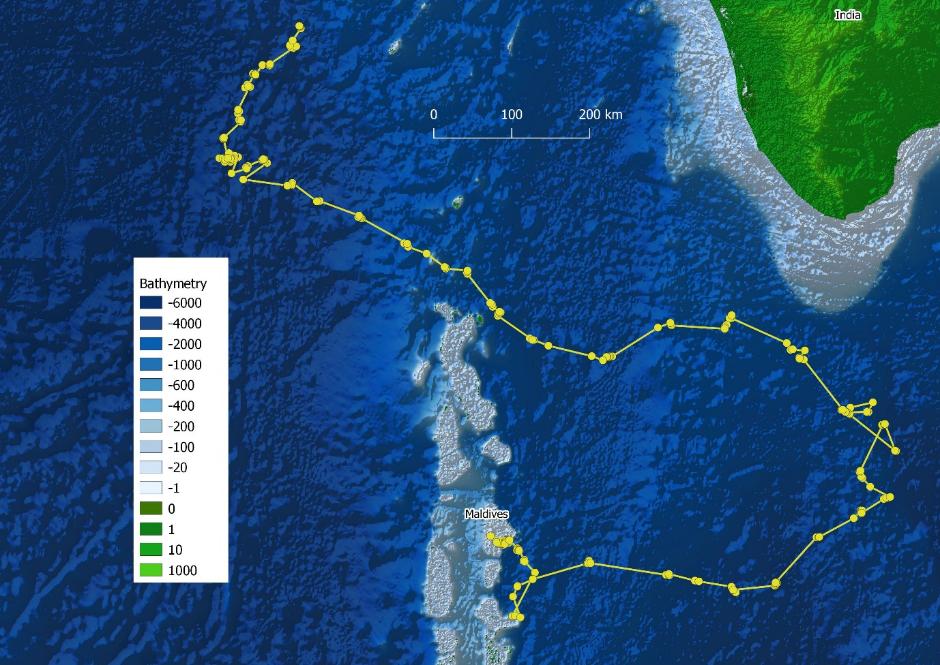


Margerita
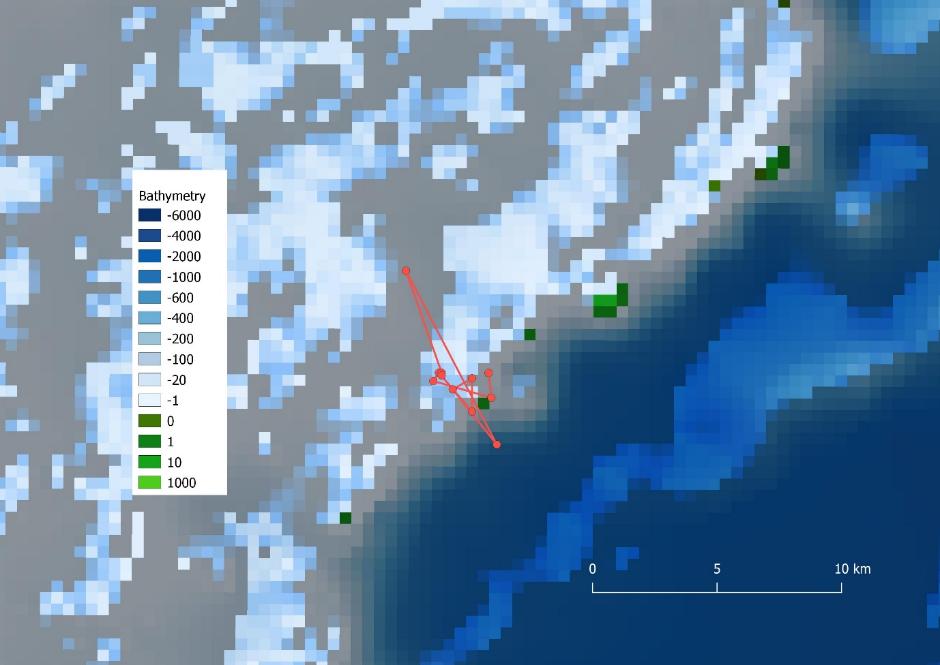


Mikka
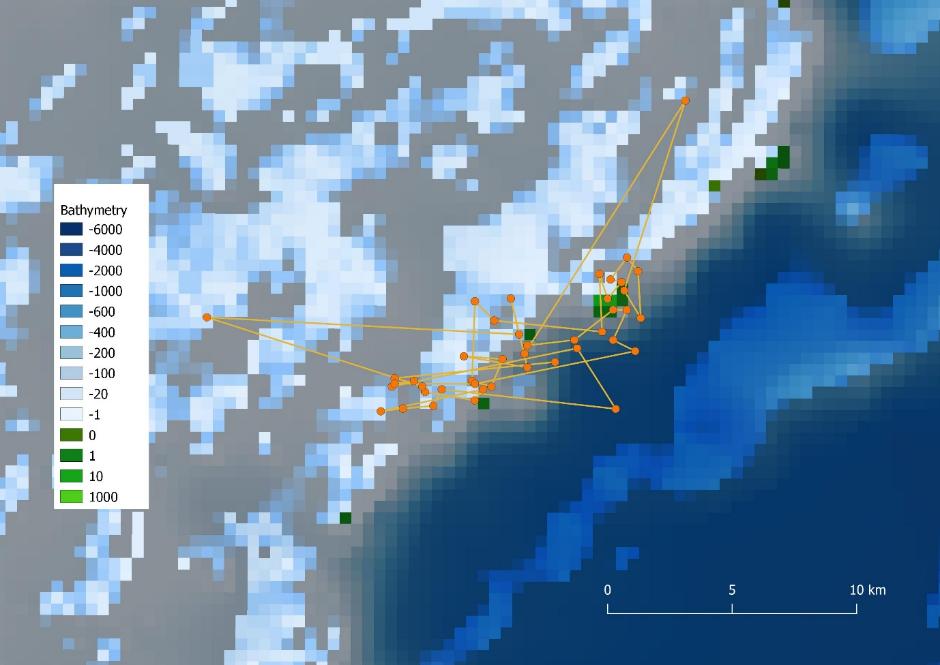


Nash
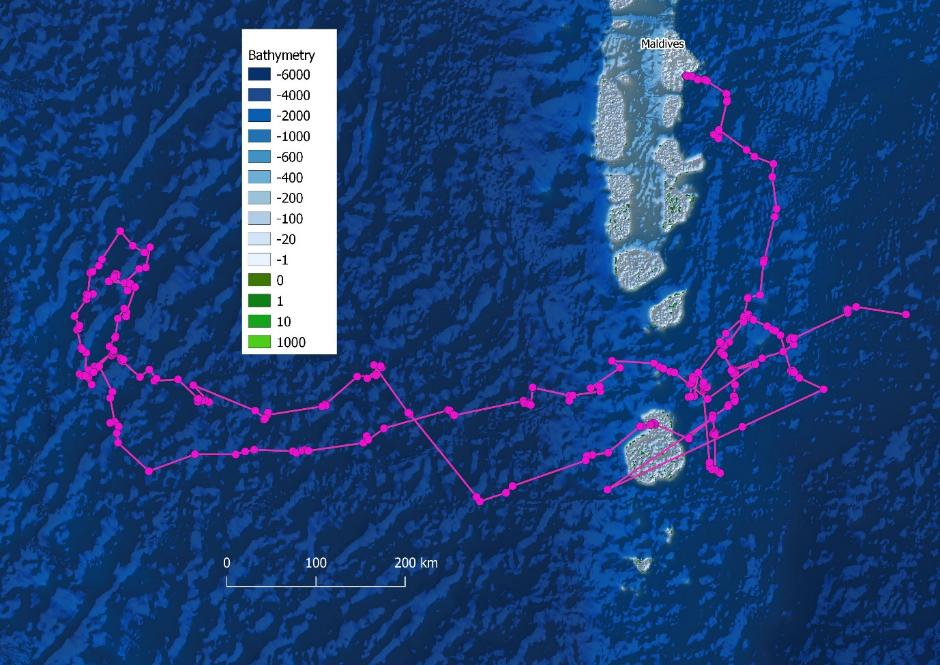


Noah
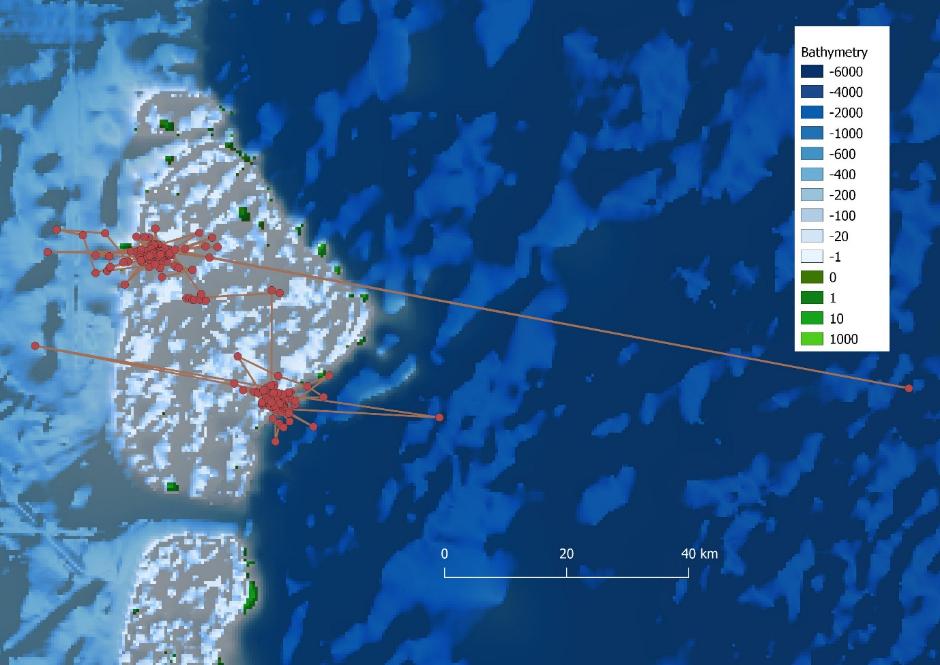


Noonu
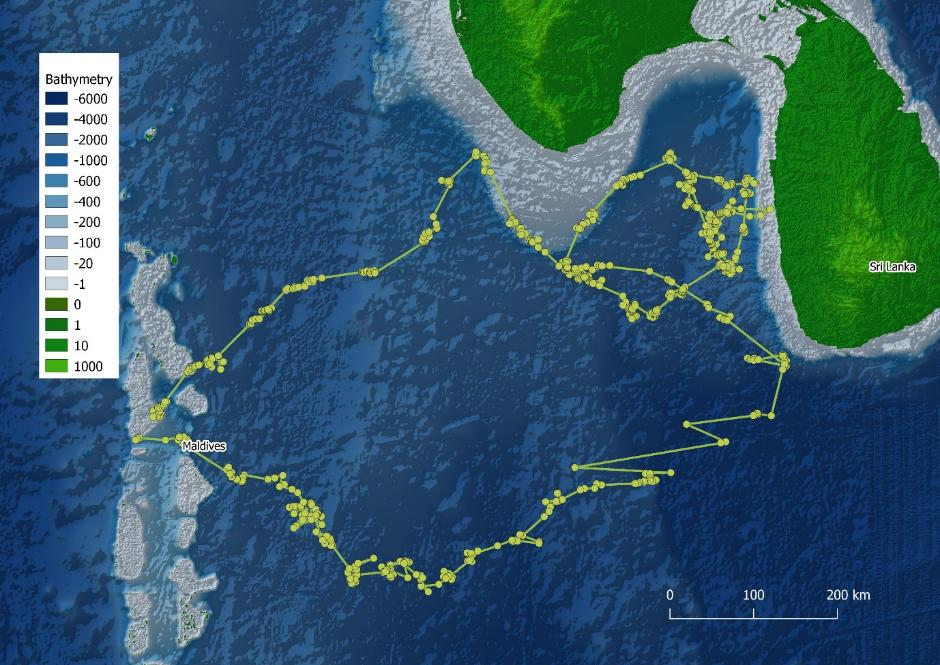


Oliver
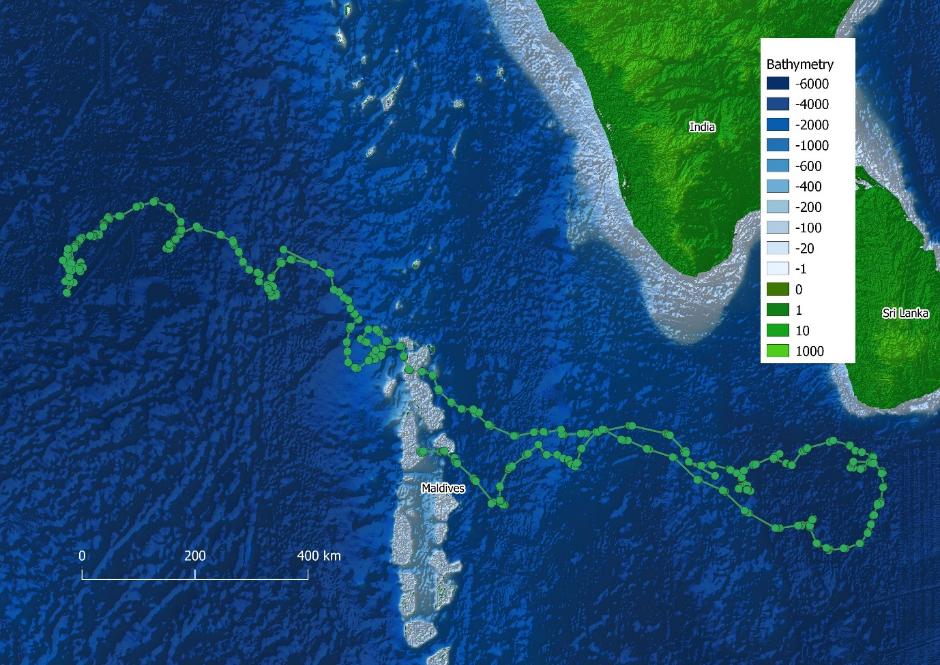


Olivia
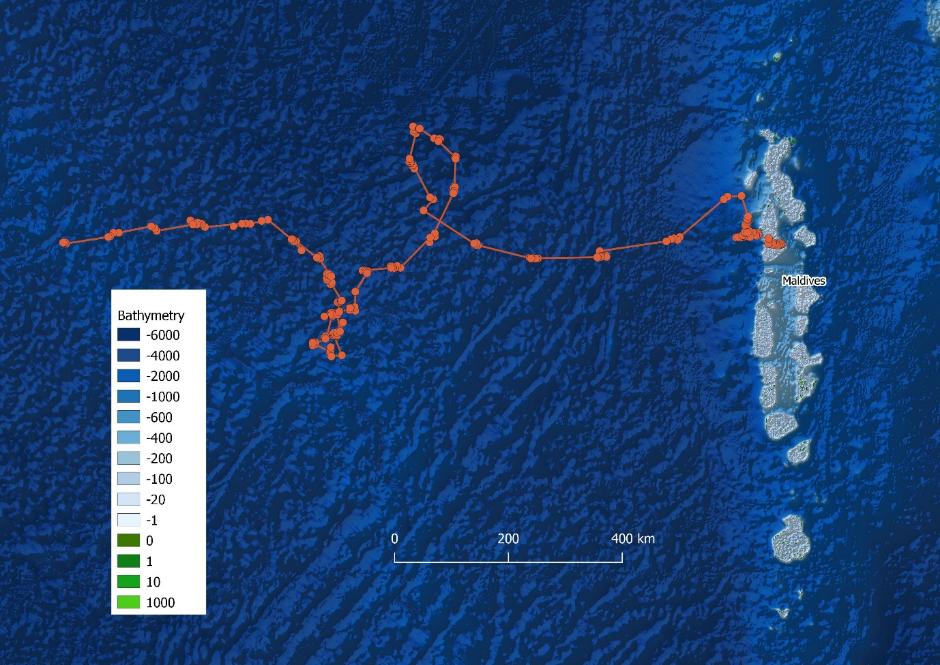


Peggy
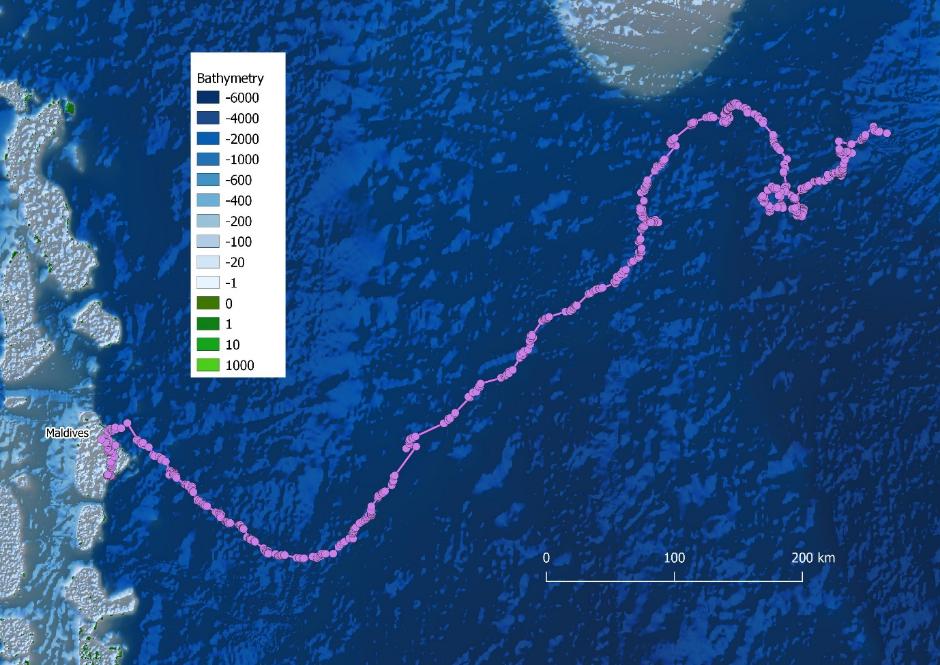


Rishi
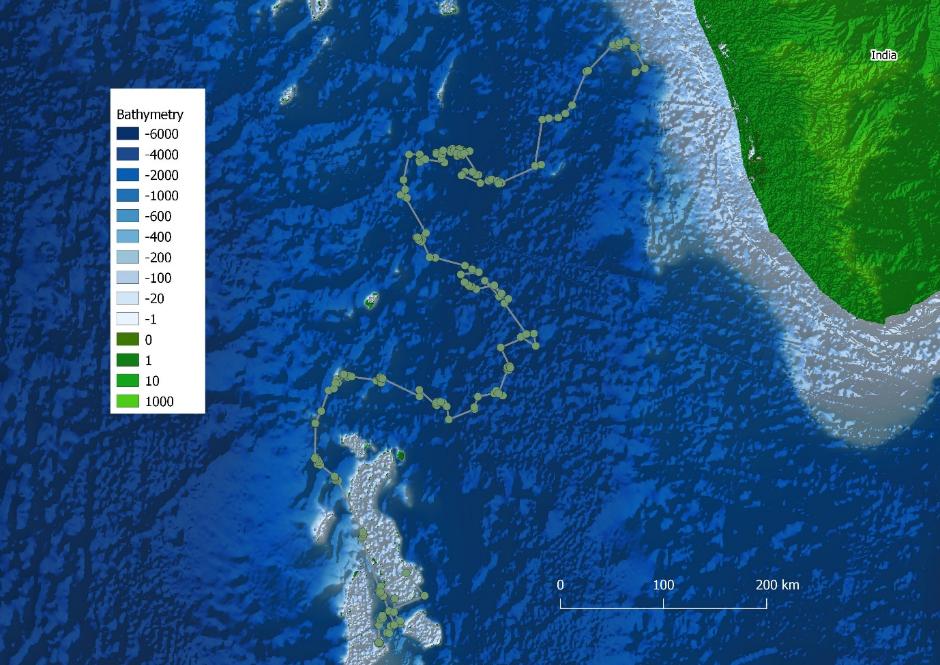


Sally
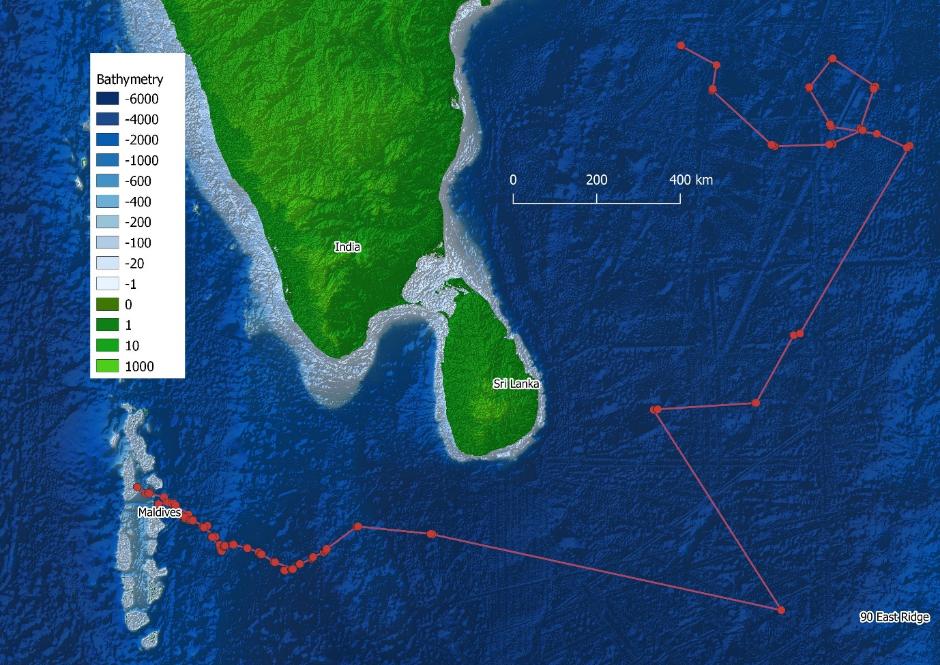


Samsy
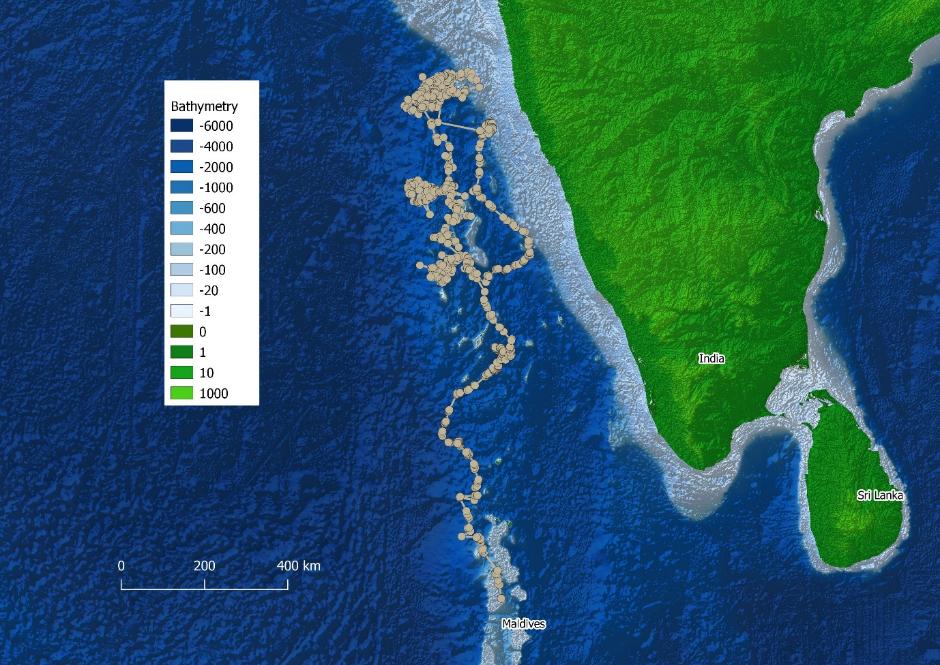


Sawan
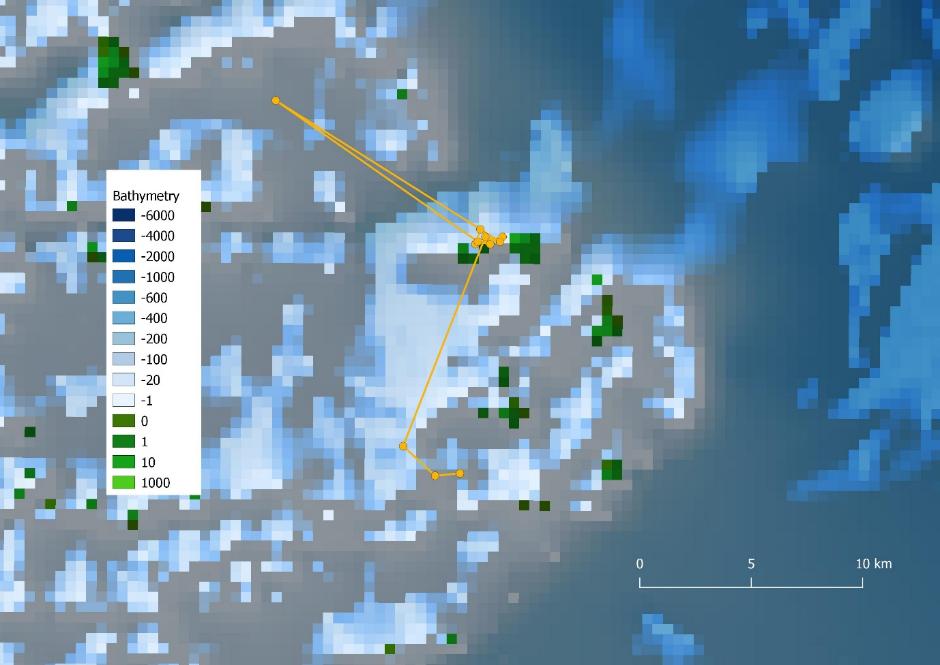


Scud
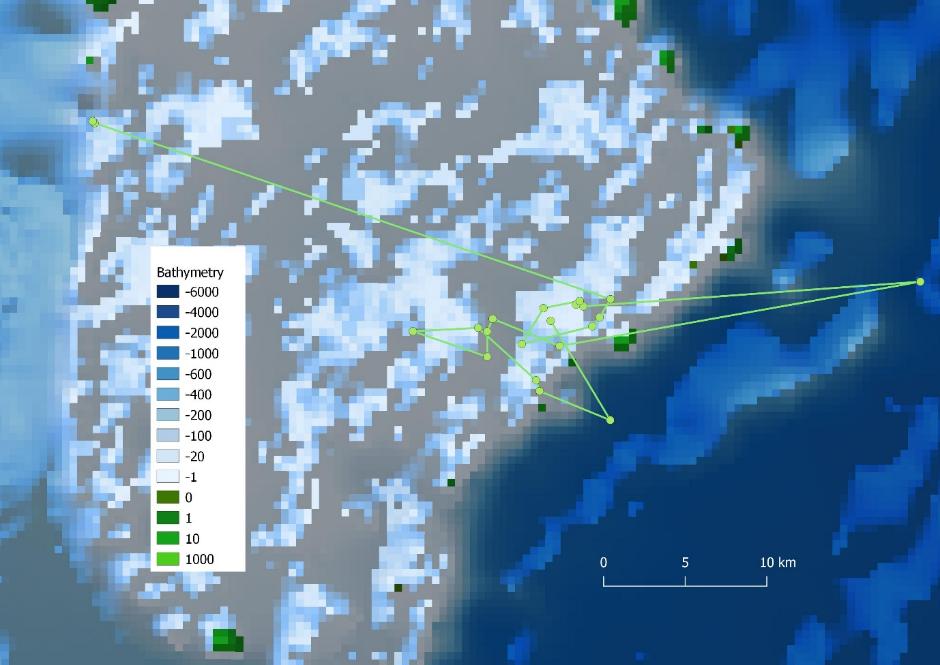


Sofia
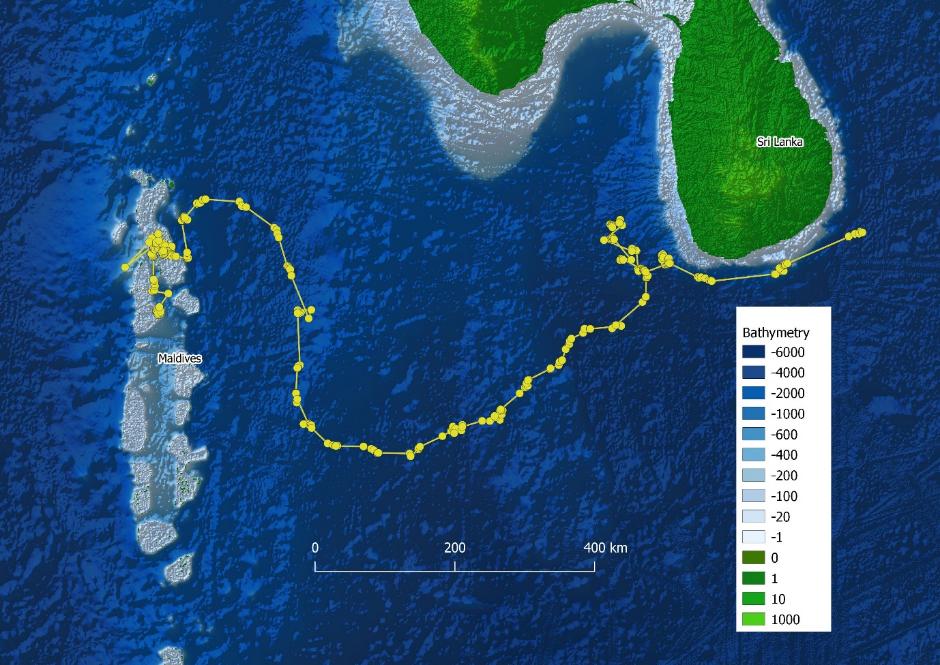


Sparticus
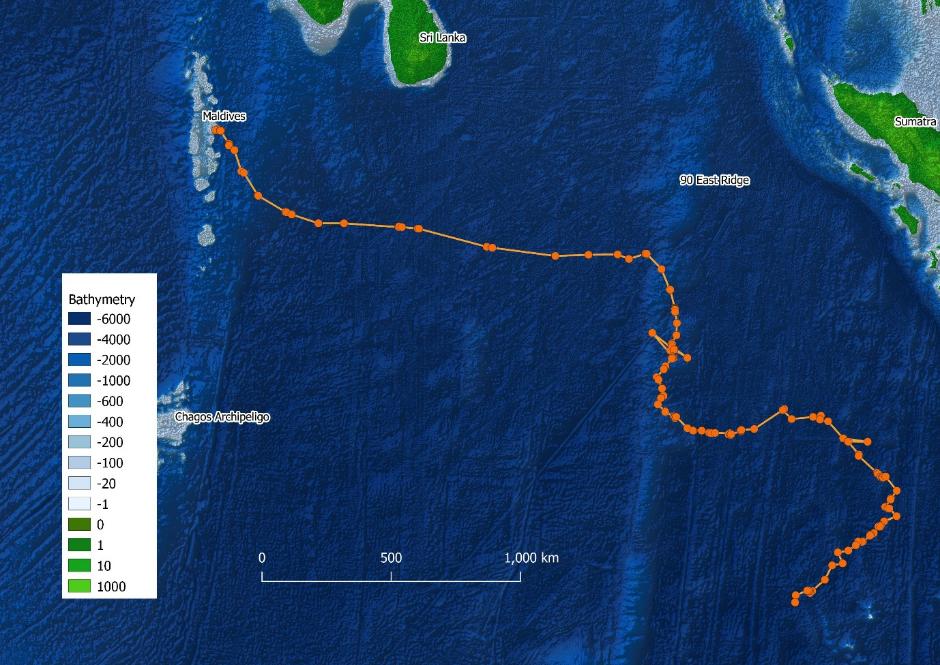


Stevie
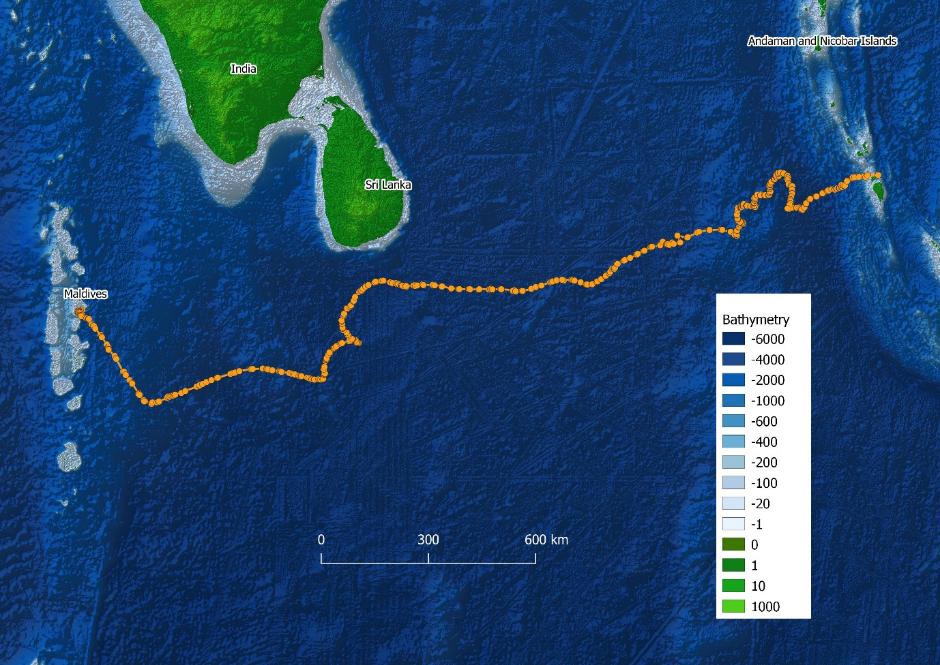


Tiger
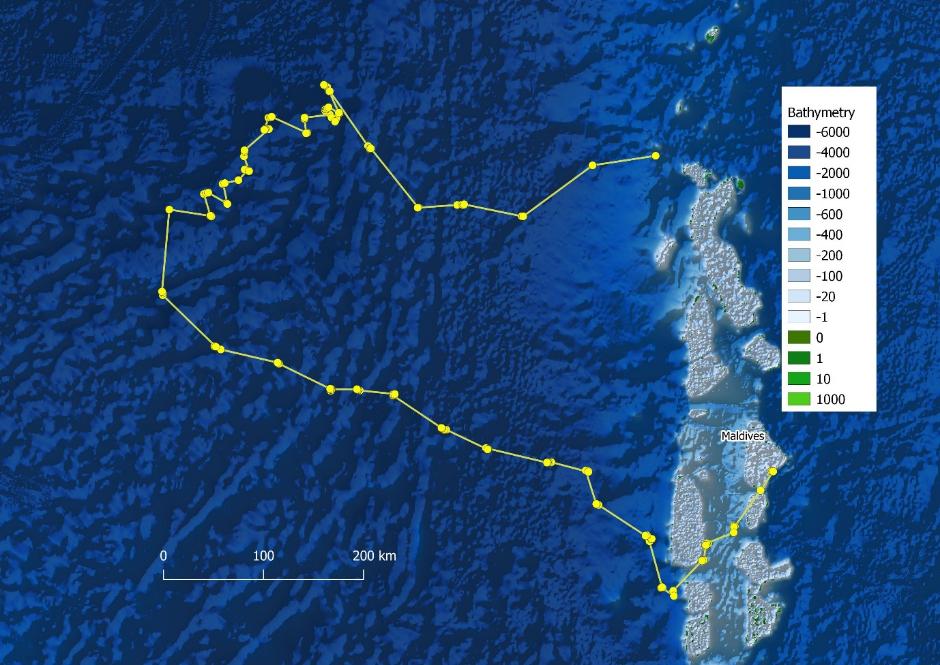


Varugadha
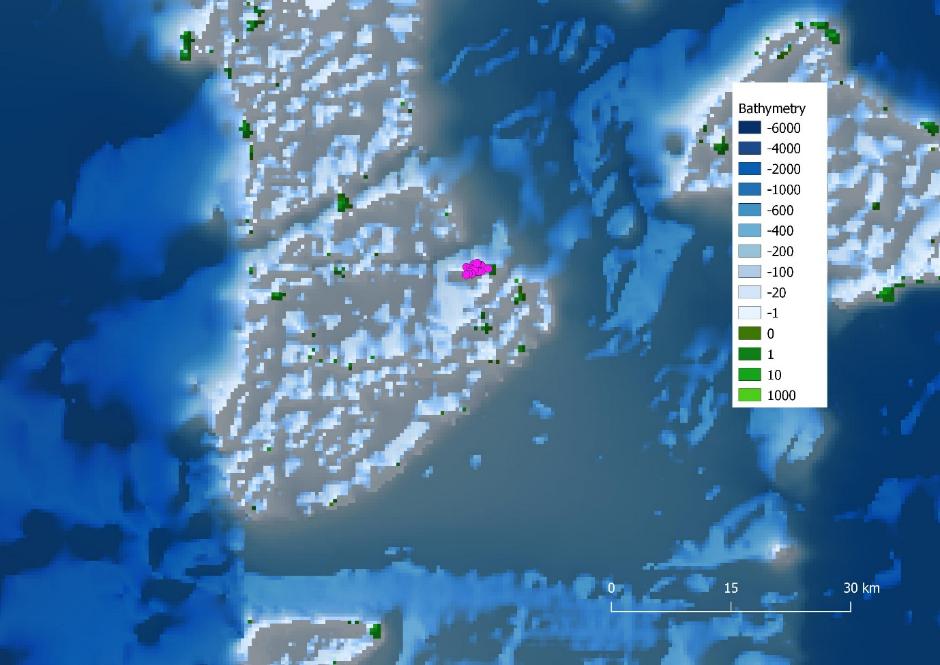


Wang
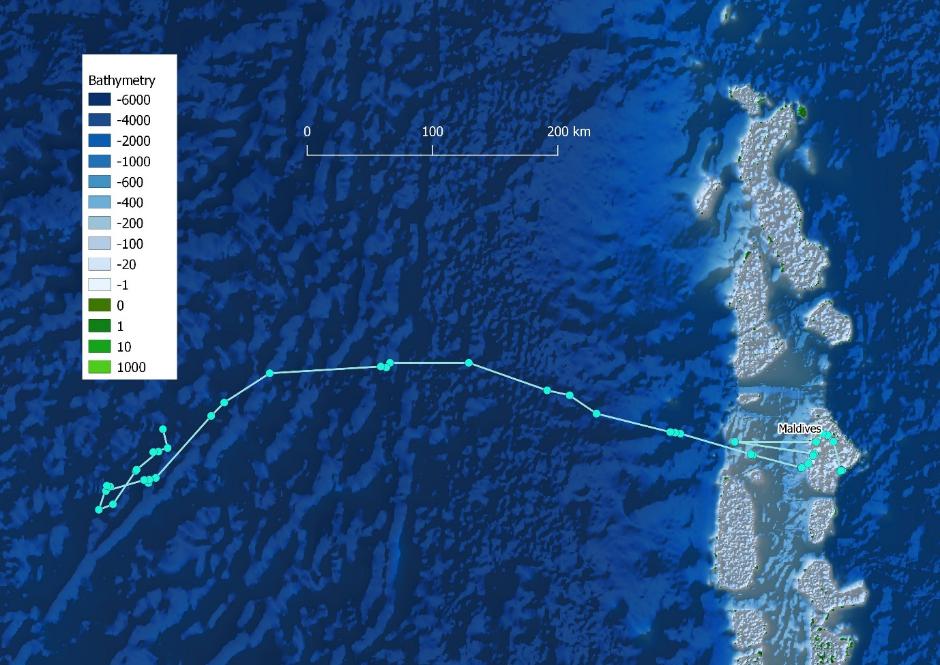

Supplement: S2 File — Figures produced by the authors in QGIS using data derived from the GEBCO 2025 Grid [49]. (DOCX) [file pone.0351541.s002.docx]
