# Supplementary figures and images for: Tracking rehabilitated sea turtles in the Indian Ocean using satellite telemetry: Insights into behaviour, ecology, and conservation implications
Source: PLoS One. 2026 Jun 26;21(6):e0351541. doi: 10.1371/journal.pone.0351541 (PMC13308851; doi:10.1371/journal.pone.0351541)

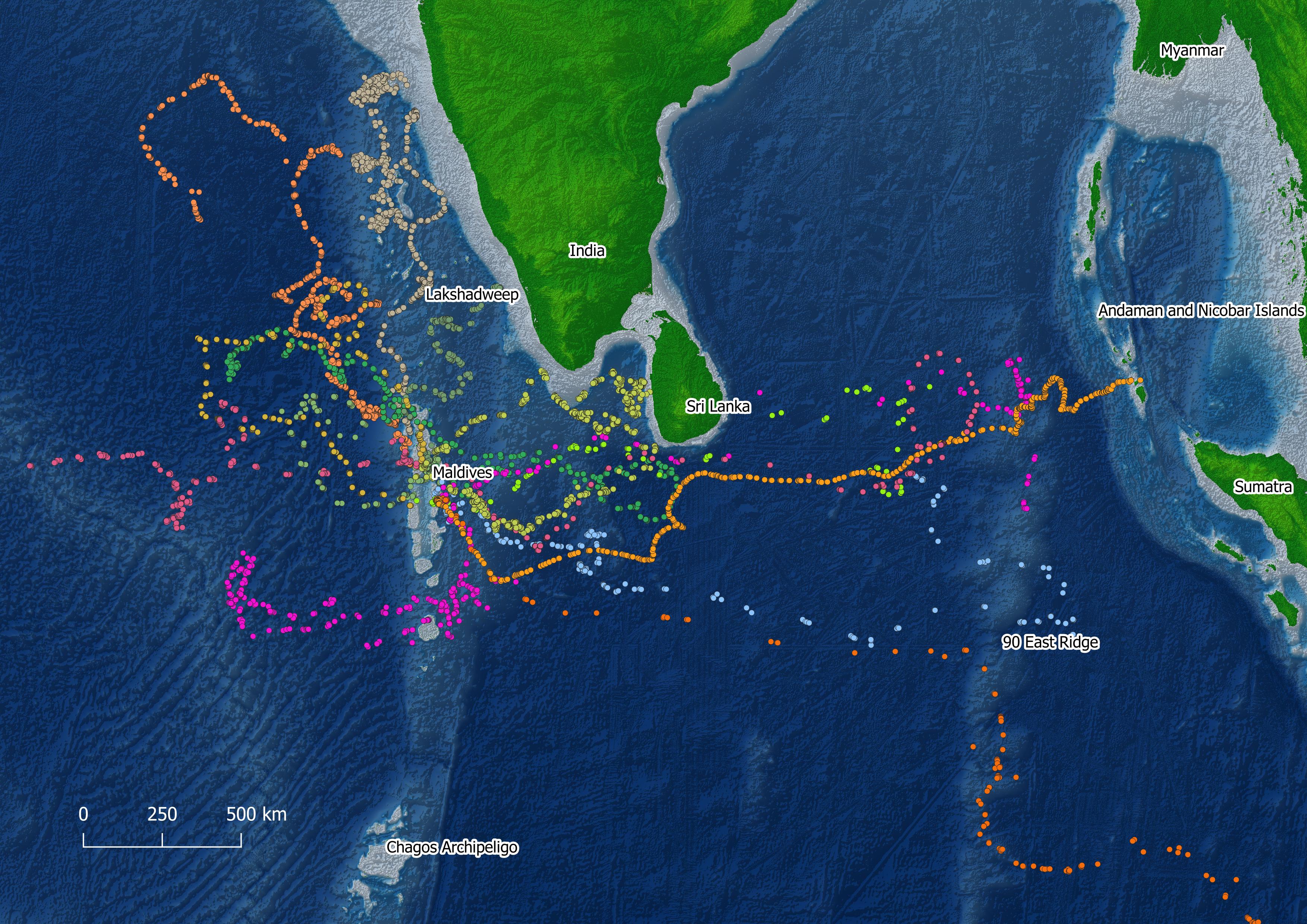

Supplement: S3 Fig — Figure produced by the authors in QGIS using data derived from the GEBCO 2025 Grid [49]. (JPEG) [file pone.0351541.s003.jpeg]
