# Supplementary material for: Tracking rehabilitated sea turtles in the Indian Ocean using satellite telemetry: Insights into behaviour, ecology, and conservation implications
Source: PLoS One. 2026 Jun 26;21(6):e0351541. doi: 10.1371/journal.pone.0351541 (PMC13308851; doi:10.1371/journal.pone.0351541)
Supplement: Attachment — Submitted filename: Response to reviewers.docx [file pone.0351541.s004.docx]

Dear reviewers,

Thank you for your constructive feedback of this manuscript. Your recommendations have been duly considered and incorporated; kindly find the responses to your comments below. Thank you for your time.

Journal Requirements:

3. We note that Figure 1 and Supplementary Information.docx in your submission contain satellite images which may be copyrighted. All PLOS content is published under the Creative Commons Attribution License (CC BY 4.0), which means that the manuscript, images, and Supporting Information files will be freely available online, and any third party is permitted to access, download, copy, distribute, and use these materials in any way, even commercially, with proper attribution. For these reasons, we cannot publish previously copyrighted maps or satellite images created using proprietary data, such as Google software (Google Maps, Street View, and Earth). For more information, see our copyright guidelines: http://journals.plos.org/plosone/s/licenses-and-copyright.

**Google Earth imagery in Figure 1 and Supplementary Information replaced with a visualisation of GEBCO_2025 Grid (Available in public domain at ‘GEBCO Compilation Group (2025) GEBCO 2025 Grid (doi:10.5285/37c52e96-24ea-67ce-e063-7086abc05f29)’)**

We require you to either (1) present written permission from the copyright holder to publish these figures specifically under the CC BY 4.0 license, or (2) remove the figures from your submission:

1. You may seek permission from the original copyright holder of Figure(s) [#] to publish the content specifically under the CC BY 4.0 license.

We recommend that you contact the original copyright holder with the Content Permission Form (http://journals.plos.org/plosone/s/file?id=7c09/content-permission-form.pdf) and the following text:

“I request permission for the open-access journal PLOS ONE to publish XXX under the Creative Commons Attribution License (CCAL) CC BY 4.0 (http://creativecommons.org/licenses/by/4.0/). Please be aware that this license allows unrestricted use and distribution, even commercially, by third parties. Please reply and provide explicit written permission to publish XXX under a CC BY license and complete the attached form.”

Please upload the completed Content Permission Form or other proof of granted permissions as an "Other" file with your submission.

In the figure caption of the copyrighted figure, please include the following text: “Reprinted from [ref] under a CC BY license, with permission from [name of publisher], original copyright [original copyright year].”

2. If you are unable to obtain permission from the original copyright holder to publish these figures under the CC BY 4.0 license or if the copyright holder’s requirements are incompatible with the CC BY 4.0 license, please either i) remove the figure or ii) supply a replacement figure that complies with the CC BY 4.0 license. Please check copyright information on all replacement figures and update the figure caption with source information. If applicable, please specify in the figure caption text when a figure is similar but not identical to the original image and is therefore for illustrative purposes only.

The following resources for replacing copyrighted map figures may be helpful:

USGS National Map Viewer (public domain): http://viewer.nationalmap.gov/viewer/

The Gateway to Astronaut Photography of Earth (public domain): http://eol.jsc.nasa.gov/sseop/clickmap/

Maps at the CIA (public domain): https://www.cia.gov/library/publications/the-world-factbook/index.html and https://www.cia.gov/library/publications/cia-maps-publications/index.html

NASA Earth Observatory (public domain): http://earthobservatory.nasa.gov/

Landsat: http://landsat.visibleearth.nasa.gov/

USGS EROS (Earth Resources Observatory and Science (EROS) Center) (public domain): http://eros.usgs.gov/#

Natural Earth (public domain): http://www.naturalearthdata.com/

4. Please include a copy of Table 1 which you refer to in your text on page 12.

**Table included in supplementary information; updated in manuscript text**

Reviewers' comments:

Reviewer's Responses to Questions

**Comments to the Author**

1. Is the manuscript technically sound, and do the data support the conclusions?

The manuscript must describe a technically sound piece of scientific research with data that supports the conclusions. Experiments must have been conducted rigorously, with appropriate controls, replication, and sample sizes. The conclusions must be drawn appropriately based on the data presented.

Reviewer #1: Yes

Reviewer #2: Yes

2. Has the statistical analysis been performed appropriately and rigorously?

Reviewer #1: N/A

Reviewer #2: Yes

3. Have the authors made all data underlying the findings in their manuscript fully available?

The [PLOS Data policy](http://www.plosone.org/static/policies.action#sharing) requires authors to make all data underlying the findings described in their manuscript fully available without restriction, with rare exception (please refer to the Data Availability Statement in the manuscript PDF file). The data should be provided as part of the manuscript or its supporting information, or deposited to a public repository. For example, in addition to summary statistics, the data points behind means, medians and variance measures should be available. If there are restrictions on publicly sharing data—e.g. participant privacy or use of data from a third party—those must be specified.

Reviewer #1: No

**Raw data included as supplementary information S2**

Reviewer #2: Yes

4. Is the manuscript presented in an intelligible fashion and written in standard English?

PLOS ONE does not copyedit accepted manuscripts, so the language in submitted articles must be clear, correct, and unambiguous. Any typographical or grammatical errors should be corrected at revision, so please note any specific errors here.

Reviewer #1: Yes

Reviewer #2: Yes

5. Review Comments to the Author

Please use the space provided to explain your answers to the questions above. You may also include additional comments for the author, including concerns about dual publication, research ethics, or publication ethics. (Please upload your review as an attachment if it exceeds 20,000 characters)

Reviewer #1: Here rehabilitated sea turtles in the Maldives were equipped with satellite tags and then tracked for several months in the Indian Ocean. The movements of three species are described as is the connectivity with other known foraging and nesting sites. In many cases the cause of cessation of tracking signals was not known.

This is a solid manuscript. Congratulations to the authors on equipping so many turtles. I think this work will make a nice contribution, but I have some suggestions for improvement.

1. Line 87. “ … migrating TO the nearby Chagos archipelago to nest …”
Probably the best paper to cite showing post-nesting green turtles returning from Chagos to the Maldives is this one: Current Biology 30, 3236–3242. <https://doi.org/10.1016/j.cub.2020.05.086>

**Text and citation updated**

2. As the reasons for the cessation of tracking is an important part of this work, I would show some plots of the diagnostic data relayed from the tags. See:
“Why do Argos satellite tags stop relaying data? Ecology and Evolution 11:7093–7101. https://doi.org/10.1002/ece3.7558”

I would show some plots of number of tag transmissions versus day of deployment to show how you assessed that battery exhaustion was not the cause of the end of tracking. Similarly show an example or two of the performance of the salt-water switch. These plots would help others to look at these diagnostic data and exclude these reasons for tag failure.

**Reasons for transmission cessation expanded upon and graphs to illustrate analysis of diagnostic data included.**

3. Immature turtles have been tracked by various groups in the Indian Ocean. Some individuals show residence to coastal areas, other take on a pelagic existence. See Fig. 2 a and b in https://doi.org/10.1002/eap.2418
See also for example:
Dominique Pelletier, David Roos, Stéphane Ciccione
Oceanic survival and movements of wild and captive-reared immature green turtles (Chelonia mydas) in the Indian Ocean. https://doi.org/10.1016/S0990-7440(03)00005-6

In your Discussion, I would briefly place your observations in the context of this other work tracking immatures in the Indian Ocean.
**Behaviour of immature turtles put into context of the wider literature during the discussion.**

4. You tracked males which is very existing. But you never mention those results. I would briefly describe if males and females showed different movements. There is an expectation that males return to breed more frequently than females. Did you see any evidence for this difference. See for example doi: 10.3389/fmars.2014.00043

**Results and discussion updated to note that there were no apparent differences in behaviour between males and females with none returning to their release sites. All adults tagged were olive ridley turtles and therefor passing vagrants rather than animals tagged during the breeding season making returning behaviour less likely to be captured.**

5. Line 265: “… one tag recorded a sudden change of direction and speed before the transmitters’ location ended in a coastal town in Sri Lanka …”
I would show a plot of these data and discuss this is good evidence for mortality. Others have used this sort of information to infer mortality. I would place your observation in the context of others who have done similar.

**Expanded upon in results and discussion.**

6. Lines 266. “… a second tag gave maximum depth readings of ~4000m beginning 5 days after the animals release and transmissions ending 20 days later.”
I do not believe these data. Note you sometimes get a blip in the transmission of data so erroneous values are obtained. Also the pressure transducer can be impacted by debris etc. and so give an anomalous reading. The tag might have relayed “max depth ever recorded”. So then the information is relayed many time so you can see if an outlier reading was just a blip. I would check. Then discuss that these values are likely erroneous so you can warn others.

**On review these readings occurred shortly before the tag stopped transmitting. Accordingly, this has been recorded as a tag error and a possible cause for the transmission ending.**

7. Lines 270. I would include a frequency distribution of length of tracking across individuals. “6 of these had a very short tracking duration (less than 6 days) …”. We’ve put on dozens of tags immature turtles in Chagos and never had such short durations of tracking. I would discuss possible reasons. Do you think these animals are quickly dying or quickly damaging the tags ?

**Point included into the discussion along with possible explanations. Graph of frequency distribution included (Figure 1)**

8. Fig. 3. Put on some axis labels. I do not know what this plot is trying to show.

**Done**

In summary, a solid manuscript that with some revisions could make a nice contribution. Graeme Hays

Reviewer #2: The manuscript is very well written, reads smoothly, and provides a substantial and highly interesting volume of data. In addition, the discussion is well structured and clearly argued. I can only offer a few minor comments, minor considerations, and one broader reflection.
Both the Results and Discussion sections indicate that, in several cases, tag transmissions were of very short duration, and in many instances it was not possible to determine the status of the animal, such as whether capture events or anomalous behavior may have occurred. Given that the study spans a relatively long time period and that tags from the same manufacturer were used throughout, and considering the growing concern within the scientific community regarding battery reliability—largely driven by global shortages associated with the rapid expansion of electric vehicles, electric scooters, and other autonomous electric transport—it would be worth exploring whether a relationship exists between the number of turtles exhibiting short transmission durations and the period in which the tags were acquired.
Specifically, it could be very informative to examine a potential correlation between transmission duration and the year of tag acquisition, in order to assess whether battery failure might be contributing to early signal loss, as has been discussed in recent scientific forums addressing tag performance issues (e.g., SCRS–ICCAT discussions on battery reliability). Alternatively, as suggested in the discussion, short transmission durations may be related to biological or environmental factors, such as tag abrasion against rocky substrates, particularly in the case of Eretmochelys imbricata (hawksbill turtle).

**Methods, results, and discussion updated to include more in-depth coverage of the reasons for end of transmission, including assessments of battery life.**

Another minor but important suggestion would be to include a summary table for each tagged individual. As the number of specimens is limited, such a table could concisely present biometric data, species identification, and key results, including transmission duration, time at liberty, mean swimming speed, and total distance traveled. This addition would greatly facilitate the reader’s interpretation of the results and enhance the overall clarity of the manuscript.

**Summary table and detailed plots of each tracked individual included in supplementary information.**

Lines 19-21: Please consider including, among other factors, habitat loss, climate change, and pollution, as these are additional drivers that may lead marine turtles toward extinction.

**Factors listed as threats to marine turtles updated in the following sentence (lines 21-24).**

6. PLOS authors have the option to publish the peer review history of their article ([what does this mean?](https://journals.plos.org/plosone/s/editorial-and-peer-review-process#loc-peer-review-history)). If published, this will include your full peer review and any attached files.


If you choose “no”, your identity will remain anonymous but your review may still be made public.


**Do you want your identity to be public for this peer review?** For information about this choice, including consent withdrawal, please see our [Privacy Policy](https://www.plos.org/privacy-policy).

Reviewer #1: No

Reviewer #2: **Yes:** Jose Carlos Báez

[NOTE: If reviewer comments were submitted as an attachment file, they will be attached to this email and accessible via the submission site. Please log into your account, locate the manuscript record, and check for the action link "View Attachments". If this link does not appear, there are no attachment files.]

To ensure your figures meet our technical requirements, please review our figure guidelines: https://journals.plos.org/plosone/s/figures

You may also use PLOS’s free figure tool, NAAS, to help you prepare publication quality figures: https://journals.plos.org/plosone/s/figures#loc-tools-for-figure-preparation.

NAAS will assess whether your figures meet our technical requirements by comparing each figure against our figure specifications.
